# Polygenic risk for mental disorder reveals distinct association profiles across social behaviour in the general population

**DOI:** 10.1038/s41380-021-01419-0

**Published:** 2022-02-28

**Authors:** Fenja Schlag, Andrea G. Allegrini, Jan Buitelaar, Ellen Verhoef, Marjolein van Donkelaar, Robert Plomin, Kaili Rimfeld, Simon E. Fisher, Beate St Pourcain

**Affiliations:** 1 Language and Genetics Department, Max Planck Institute for Psycholinguistics, Wundtlaan 1, 6525 XD Nijmegen, The Netherlands; 2 Social, Genetic and Developmental Psychiatry Centre, Institute of Psychiatry, Psychology & Neuroscience, King’s College London, Memory Ln, Camberwell, London, SE5 8AF London, UK; 3 Psychology and Language Sciences, University College London, 26 Bedford Way, Bloomsbury, London, WC1H 0AP London, UK; 4 Donders Institute for Brain, Cognition and Behaviour, Radboud University, Kapittelweg 29, 6525 EN Nijmegen, The Netherlands; 5 Karakter Child and Adolescent Psychiatry University Centre, Reinier Postlaan 12, 6525 GC Nijmegen, The Netherlands; 6 Department of Cognitive Neuroscience, Radboud University Medical Center, Geert Grooteplein 21, 6525 EZ Nijmegen, The Netherlands; 7 MRC Integrative Epidemiology Unit, University of Bristol, Oakfield House, Oakfield Grove, Bristol, BS8 2BN UK; 8 Population Health Sciences, University of Bristol, 5 Tyndall Avenue, Bristol, BS8 1UD UK

**Keywords:** Psychiatric disorders, Genetics, Psychology

## Abstract

Many mental health conditions present a spectrum of social difficulties that overlaps with social behaviour in the general population including shared but little characterised genetic links. Here, we systematically investigate heterogeneity in shared genetic liabilities with attention-deficit/hyperactivity disorder (ADHD), autism spectrum disorders (ASD), bipolar disorder (BP), major depression (MD) and schizophrenia across a spectrum of different social symptoms. Longitudinally assessed low-prosociality and peer-problem scores in two UK population-based cohorts (4–17 years; parent- and teacher-reports; Avon Longitudinal Study of Parents and Children(ALSPAC): *N* ≤ 6,174; Twins Early Development Study(TEDS): *N* ≤ 7,112) were regressed on polygenic risk scores for disorder, as informed by genome-wide summary statistics from large consortia, using negative binomial regression models. Across ALSPAC and TEDS, we replicated univariate polygenic associations between social behaviour and risk for ADHD, MD and schizophrenia. Modelling variation in univariate genetic effects jointly using random-effect meta-regression revealed evidence for polygenic links between social behaviour and ADHD, ASD, MD, and schizophrenia risk, but not BP. Differences in age, reporter and social trait captured 45–88% in univariate effect variation. Cross-disorder adjusted analyses demonstrated that age-related heterogeneity in univariate effects is shared across mental health conditions, while reporter- and social trait-specific heterogeneity captures disorder-specific profiles. In particular, ADHD, MD, and ASD polygenic risk were more strongly linked to peer problems than low prosociality, while schizophrenia was associated with low prosociality only. The identified association profiles suggest differences in the social genetic architecture across mental disorders when investigating polygenic overlap with population-based social symptoms spanning 13 years of child and adolescent development.

## Introduction

Many heritable mental disorders such as attention-deficit/hyperactivity disorder (ADHD), autism spectrum disorders (ASD), bipolar disorder (BP), major depression (MD) or schizophrenia are characterised by social-behavioural difficulties. In ADHD, these predominantly include peer problems [1], while ASD is characterised by deficits in social interaction and communication [2] that may, in turn, lead to risk of being bullied [3]. Individuals with BP can suffer from social withdrawal and poor social functioning [4], and, similarly, those with MD may show social withdrawal and disrupted social processing [5]. Individuals with schizophrenia often have poor social cognition and lack social interest [6].

The underlying social-behavioural difficulties can be diverse. They may reflect a lack of positive interactions involving low prosocial behaviour reflected in limited helping, sharing and cooperating with others [7]. Alternatively, peer problems describe problematic interactions such as social withdrawal, being bullied, and the inability to get along with others [8]. One of the grand challenges in psychiatric genetics is to understand how common genetic risk can manifest as a spectrum of diverse symptoms. Genome-wide efforts in large consortia have demonstrated the single-nucleotide polymorphism-based heritability (SNP-h^2^) of ADHD (0.22) [9], ASD (0.11) [10], BP (0.18) [11], MD (0.09) [12] and schizophrenia (0.22; Supplementary Table 1) [13]. Genetic overlap between social cognition-/social communication-related abilities and mental disorders [14–17], including neurodevelopmental conditions [15–19], suggests that also social-behavioural symptoms in psychopathology may represent an underlying dimension that is shared with social traits in the general population.

Social behaviour is known to be heritable. Twin studies have reported heritability estimates of 0.38–0.76 [20–22] for prosocial behaviour and 0.41–0.83 [20, 23] for peer problems. Consistent with social symptom changes throughout development and across different social situations [24], there is variation in genetic influences across developmental stages [22, 23], social environment as reported by teachers or parents [20, 21] and social traits [20] in population-based samples. Heritability estimates as captured by SNPs range between 0.02 and 0.27 for parent-reported peer problems in the general population, with larger estimates during adolescence compared to childhood [23], strengthening the evidence for developmental changes in genetic architectures. Thus, given the genetic heterogeneity in social behaviour, also polygenic links with disorder may systematically vary across the spectrum of social behaviour.

In this open science framework registered study (https://osf.io/p5wah/) [25], we systematically investigate genetic links between mental disorders, as informed by genome-wide summary statistics from large consortia, and child and adolescent social behaviour in the general population, studying heterogeneity in polygenic associations across different ages, reporters and social traits, adopting a two-stage research design:

Within stage 1, we assess the relationship of polygenic risk scores (PRS) for ADHD, ASD, BP, MD, and schizophrenia risk with population-based low-prosociality and peer-problem scores (Strengths-and-Difficulties questionnaire (SDQ) subscales [26], age 7–17 years, parent- and teacher-reports) in the UK Avon Longitudinal Study for Parents and Children (ALSPAC) [27]. We follow up findings with matching PRS and SDQ social scores (age 4–16 years; parent- and teacher-reports) in the UK Twins Early Development Study (TEDS) [28].

Within stage 2, we model heterogeneity in polygenic associations as predicted by age-, reporter-, and trait-specific social-behavioural (SDQ) measures. We combine univariate findings from ALSPAC and TEDS using a mixed-effects meta-regression approach and identify and compare social-behavioural association patterns across disorders.

## Samples and methods

### Genome-wide summary statistics for mental disorder

We studied genome-wide summary statistics for five mental disorders as published by the Psychiatric Genomic Consortium (PGC), the Danish Lundbeck Foundation Initiative for Integrative Psychiatric Research (iPSYCH) and/or the UK Biobank (UKBB): ADHD-PGC/iPSYCH [9], ASD-PGC/iPSYCH [10], BP-PGC [11], MD-PGC/UKBB [12], and schizophrenia-PGC [13]. Cohort details including ancestry, size, imputation reference panel, symptoms and age-of-onset of the disorder are described in the Supplementary Methods and Supplementary Table 1.

### Social behaviour in the general population

ALSPAC is a UK population-based longitudinal pregnancy-ascertained birth cohort with birth dates between 1991 and 1992 [27, 29]. Ethical approval for the study was obtained from the ALSPAC Ethics and Law Committee and the Local Research Ethics Committees. Consent for biological samples has been collected in accordance with the Human Tissue Act (2004). Informed consent for the use of data collected via questionnaires and clinics was obtained from participants following recommendations of the ALSPAC Ethics and Law Committee at the time (Supplementary Methods).

TEDS is a population-based longitudinal study of >10,000 twin pairs representative of England and Wales, recruited from 1994 to 1996 births [28]. Ethical approval for the study was granted by King’s College London’s ethics committee for the Institute of Psychiatry, Psychology and Neuroscience (05.Q0706/228), and written informed consent was given by the parents prior to data collection.

Phenotype information: Prosocial behaviour and peer problems were assessed in ALSPAC and TEDS children (Supplementary Methods; Table 1). Both, prosocial behaviour (here recoded as low-prosociality scores) and peer problems were assessed using subscales of the SDQ [26], based on parent- and teacher-reports at the same ages. In ALSPAC, parent-reported (predominantly mother-reported) behaviour was measured at the ages of 7, 10, 12, 13, and 17 years and in TEDS at the ages of 4, 7, 9, 11, and 16 (prosocial scores only) years. In addition, teacher reports were obtained at the ages of 8 and 11 years in ALSPAC and at the ages of 7, 9 and 12 years in TEDS. Phenotypically, both scores are modestly to moderately correlated with each other (Supplementary Tables 2, 3).

**Table 1:** Descriptive information of low-prosociality and peer-problem scores in ALSPAC and TEDS.

		Age (years)	Variable score		
		Mean (SD)	Mean (SD)	% Males	N
*ALSPAC*					
Low prosociality^a^					
Parent-reported:	7Y	6.79 (0.11)	1.82 (1.75)	51	5,610
	10Y	9.65 (0.12)	1.66 (1.65)	50	5,670
	12Y	11.72 (0.13)	1.65 (1.68)	50	5,268
	13Y	13.16 (0.18)	2.76 (1.73)	50	5,069
	17Y	16.84 (0.36)	1.97 (1.87)	48	4,151
Teacher-reported:	8Y	8.33 (0.31)	2.21 (2.42)	50	3,686
	11Y	11.16 (0.33)	2.06 (2.35)	50	4,417
Peer problems					
Parent-reported:	7Y	6.79 (0.11)	1.02 (1.04)	51	5,608
	10Y	9.65 (0.12)	1.10 (1.49)	50	5,661
	12Y	11.72 (0.13)	1.10 (1.56)	50	5,263
	13Y	13.16 (0.18)	1.19 (1.61)	50	5,061
	17Y	16.84 (0.36)	1.11 (1.51)	48	4,156
Teacher-reported:	8Y	8.33 (0.31)	1.13 (1.74)	50	3,689
	11Y	11.16 (0.33)	1.20 (1.85)	50	4,417
*TEDS*					
Low prosociality^a^					
Parent-reported:	4Y	4.04 (0.12)	2.60 (1.86)	48	6,958
	7Y	7.06 (0.25)	1.84 (1.79)	48	7,112
	9Y	9.01 (0.29)	2.71 (1.71)	47	3,375
	11Y	11.25 (0.7)	1.46 (1.65)	48	6,039
	16Y	16.31 (0.68)	1.74 (1.94)	45	5,252
Teacher-reported:	7Y	7.20 (0.28)	2.68 (2.36)	49	5,900
	9Y	9.03 (0.29)	2.44 (2.26)	47	2,825
	12Y	11.50 (0.66)	1.99 (2.09)	47	4,931
Peer problems					
Parent-reported:	4Y	4.04 (0.12)	1.52 (1.54)	48	6,948
	7Y	7.06 (0.25)	1.01 (1.45)	48	7,112
	9Y	9.01 (0.29)	1.11 (1.59)	47	3,370
	11Y	11.25 (0.7)	1.11 (1.54)	48	6,023
Teacher-reported:	7Y	7.20 (0.28)	1.07 (1.48)	49	5,900
	9Y	9.03 (0.29)	0.85 (1.47)	47	2,828
	12Y	11.51 (0.66)	1.04 (1.6)	47	4,964

All low-prosociality and peer-problem scores were assessed using the Strengths-and-Difficulties questionnaire.

*ALSPAC* Avon Longitudinal study of Parents and Children, *SDQ* Strengths-and-Difficulties questionnaire, *TEDS* Twins Early Development Study, *Y* Age in years.

^a^Reverse coded SDQ prosocial scale.

### Univariate polygenic scoring analyses in ALSPAC and TEDS

Polygenic scoring analyses: Consistent with current guidelines [30], we constructed PRS for each disorder (ADHD, ASD, BP, MD and schizophrenia) within ALSPAC and TEDS using a clumping and thresholding approach (PRS(C + T); nine risk-variant selection thresholds 0.001 ≤ *P*_T_ < 1), based on high-quality genome-wide imputed SNPs (Supplementary Methods).

Within ALSPAC, we studied unrelated children and adolescents (genomic relatedness < 0.125). We regressed untransformed social-behavioural scores (peer problems or low prosociality) on Z-standardised PRS using a negative binomial model (R:MASS; Supplementary Methods). PRS effects (β) were adjusted for sex, age, and the first two principal components (PCs). As part of cross-disorder adjusted analyses, disorder PRS effects were also corrected for each other. Within TEDS, we analysed pairs of dizygotic twins and a single twin of each monozygotic pair. PRS association analyses were conducted using a mixed-effects negative binomial regression approach (R:lme4.v.1.1-26 [31]) with a random intercept to adjust for family relatedness and fixed effects for PRS adjusted for sex, age, the first ten PCs, genotyping-batch, genotyping-chip effects, and, if cross-disorder adjusted, also other disorder PRS. For both the negative binomial and the mixed-effects negative binomial model, β indicates the change in log counts of the social score by one SD change in PRS. We tested the predictive ability of PRS using ΔMcFadden’s-R^2^ (Supplementary Methods) [32].

For sensitivity analyses, we repeated PRS analyses in ALSPAC using PRS-CS [33], a method that applies a continuous-shrinkage parameter to adjust SNP effect sizes for linkage disequilibrium (Supplementary Methods).

Multiple-testing correction: Using Matrix Spectral Decomposition (matSpD) [34], we adjusted the multiple-testing burden of univariate PRS analyses in ALSPAC across the 14 interrelated social-behavioural scores for an effective number of 10 independent variables (Supplementary Table 2) and five disorder PRS to 0.05/(10 × 5) = 0.001. For follow-up analyses in TEDS, with an effective number of 12 independent variables, the multiple-testing burden under a one-sided test was adjusted to 0.1/(12 × 5) = 0.0017, accounting for 15 interrelated scores (Supplementary Table 3) and five disorder PRS.

Power analyses: We estimated covariance and power (R:avengeme [35]) to detect effects across all studied PRS(C + T) *P*-value thresholds in the discovery cohort (ALSPAC; Supplementary Methods).

### Meta-regression of polygenic effects

Meta-regression models: For each disorder, we combined univariate PRS(C + T) effects across ALSPAC and TEDS using a mixed-effects meta-regression model (R:metafor.v.2.1-0 [36], Supplementary Methods). Univariate PRS(C + T) effects were based on a representative risk variant selection threshold of *P*_T_ ≤ 0.1 (Supplementary Fig. 1). In brief, we systematically assessed whether heterogeneity in PRS association effects can be attributed to differences in social behaviour explained by the median age of assessment, reporter (parent versus teacher), and SDQ-based social trait (low prosociality versus peer problems). For each disorder, we fitted a full model including a random intercept accounting for repeated measures (nested within each cohort) as well as fixed effects for age-, reporter-, trait- and/or cohort-specific effects. The most parsimonious model was identified by dropping successively fixed effects from the model (likelihood-ratio test at *P* > 0.05) and assessing residual heterogeneity (Cochran’s-Q test; Supplementary Methods). The inter-relatedness of PRS association effects across SDQ-based social measures within each cohort was accounted for by constructing a composite variance-covariance matrix analogous to models accounting for correlated phylogenetic histories [37]. For sensitivity analyses, we also compared combinations of univariate PRS(C + T) effects with univariate PRS-CS effects in ALSPAC only.

Multiple-testing correction: A threshold of *P* ≤ 0.01 (0.05/five disorders) was applied.

### Biological-pathway-based PRS analyses

To study biological processes underlying univariate PRS effects conditional on variants selected at *P*_T_ ≤ 0.1, we extended the PRS(C + T) approach with exploratory PRSet [38] analyses in ALSPAC (not preregistered). Defining for each disorder a baseline at *P*_T_ ≤ 0.1, we constructed subsets of pathway-PRS for 7,481 gene sets based on gene ontology biological pathways (GOBP; Supplementary Methods). Using the same negative binomial regression framework as for PRS(C + T) analyses, we investigated for each disorder genetic links between pathway-PRS and social behaviour, focussing on measures with the strongest meta-analytic evidence for association. To control for inflated type I error, we screened for pathway-PRS that reached the same strength of association as baseline-PRS and passed the multiple-testing threshold in ALSPAC (*P*_pathway_ ≤ *P*_baseline_ ≤ 0.001).

## Results

### Stage 1: Univariate association analyses

**Fig. 1:**
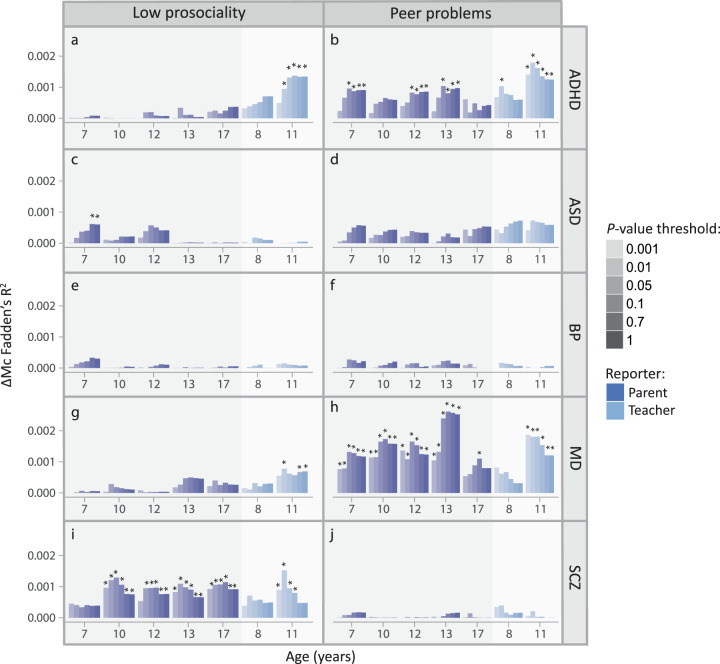
Association between PRS(C + T) for mental disorder and social behaviour in ALSPAC. ΔMcFadden’s-R^2^ is shown for the prediction of low-prosociality and peer-problem scores by ADHD-PRS (**a**, **b**), ASD-PRS (**c**, **d**), BP-PRS (**e**, **f**), MD-PRS (**g**, **h**), SCZ-PRS (**i**, **j**). Mental disorder genome-wide summary statistics (ADHD-PGC/iPSYCH, ASD-PGC/iPSYCH, BP-PGC, MD-PGC/UKBB, and SCZ-PGC) were used to construct Z-standardised PRS(C + T) in ALSPAC (ADHD-PRS, ASD-PRS, BP-PRS, MD-PRS, and SCZ-PRS) at multiple *P-*value thresholds. Association analyses with social behaviour (low-prosociality and peer-problem scores) were conducted using negative binomial regression (non-adjusted for cross-disorder PRS effects; multiple-testing corrected *P*-value: *P ≤ 0.001). ADHD Attention-deficit/hyperactivity disorder, ALSPAC Avon Longitudinal study of Parents and Children, ASD Autism spectrum disorders, BP Bipolar disorder, C + T clumping and thresholding, iPSYCH Lundbeck Foundation Initiative for Integrative Psychiatric Research, MD Major depression, PGC Psychiatric Genomics consortium, PRS Polygenic risk scores, *P*_T_ PRS *P*-value threshold, SCZ Schizophrenia. Low-prosociality and peer-problem scores were assessed using the Strengths-and-Difficulties questionnaire.

Discovery analyses in ALSPAC: We assessed univariate associations between each of the 14 population-based social-behavioural scores in ALSPAC, including low-prosociality and peer-problem scores between the ages of 7 and 17 years as reported by parents or teachers, and five disorder-PRS(C + T) related to ADHD, ASD, BP, MD, and schizophrenia risk (multiple-testing threshold: *P* ≤ 0.001). All social scores were skewed, with most children showing few difficulties in prosocial behaviour and peer interactions (Table 1). Given a better model fit, we studied genetic associations with negative binomial regressions (Supplementary Table 4). PRS effects were estimated across nine variant selection thresholds (0.001 ≤ *P*_T_ < 1; Supplementary Tables 5, 6; Fig. 1), but are here, for simplicity, reported at *P*_T_ ≤ 0.1.

Many social-behavioural scores were associated with polygenic risk for ADHD, MD and schizophrenia. For ADHD-PRS, the strongest association was identified for teacher-reported peer problems at the age of 11 years (β_ADHD_11Y_(SE) = 0.10(0.025), ΔMcFadden’s-R^2^ = 0.0013, *P* = 2.5 × 10^−5^; Fig. 1a, b). MD-PRS was most strongly associated with parent-reported peer problems scores at 13 years (β_MD_13Y_(SE) = 0.12(0.019), ΔMcFadden’s-R^2^ = 0.0026, *P* = 2.6 × 10^−10^; Fig. 1g, h). Associations between schizophrenia-PRS and social traits were strongest for teacher-rated low-prosociality scores at 11 years (β_SCZ_11Y_(SE) = 0.07(0.019), ΔMcFadden’s-R^2^ = 8.0 × 10^−4^, *P* = 2.2 × 10^−4^; Fig. 1i, j). For ASD-PRS, no univariate association with social symptoms at *P*_T_ ≤ 0.1 passed the multiple-testing threshold. However, at less stringent *P*_T_ thresholds, association with parent-reported low prosociality at seven years was present (e.g. at *P*_T_ < 0.5, β_ASD_7Y_(SE) = 0.045(0.013), ΔMcFadden’s-R^2^ = 5.8 × 10^−4^, *P* = 6.6 × 10^−4^; Fig. 1c, d). There was little evidence for association between BP-PRS and any of the studied social measures (Fig. 1e, f).

PRS(C + T) power analyses (Supplementary Fig. 2) showed that, across all studied mental health conditions, our study had sufficient power under the assumption of fixed trait-disorder covariance (equivalent to the SNP-h^2^ of the disorder; Supplementary Table 7). Once data-driven trait-disorder covariance and, thus, trait architectures (Supplementary Table 8) were taken into consideration, power curves followed observed association patterns. Here, the power to detect polygenic overlap with BP risk was consistently low (<80%). This suggests that changes in association effects are likely to reflect changes in genetic overlap between trait and disorder rather than differential power in disorder PRS due to a lack of SNP-h² of the disorder genome-wide association study (GWAS) discovery sample. The estimated genetic trait-disorder covariance at *P*_T_ < 0.1 was largely representative across the range of studied *P*-value thresholds (Supplementary Fig. 1).

Sensitivity analyses using alternative polygenic scoring methods (PRS-CS) confirmed the identified univariate association patterns (Supplementary Fig. 3; Supplementary Tables 9, 10).

**Fig. 2:**
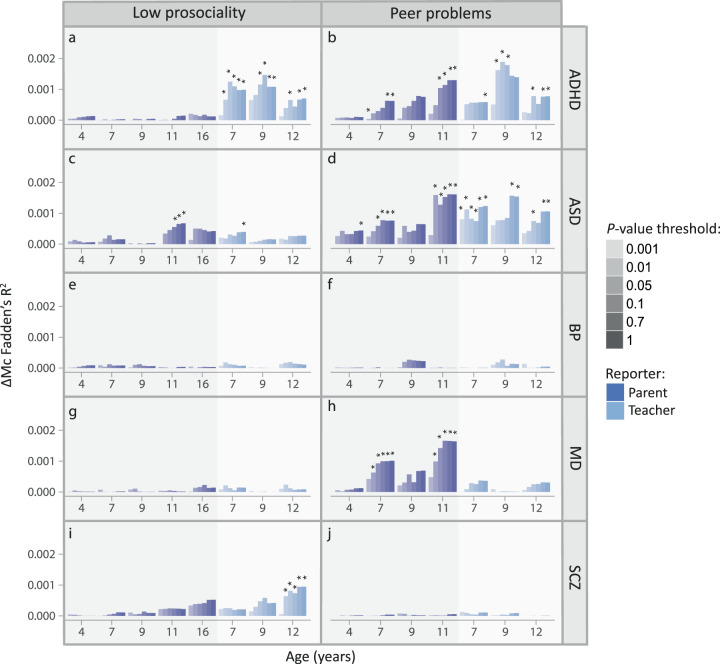
Association between PRS(C + T) for mental disorder and social behaviour in TEDS. ΔMcFadden’s-R^2^ is shown for the prediction of low-prosociality and peer-problem scores by ADHD-PRS (**a**, **b**), ASD-PRS (**c**, **d**), BP-PRS (**e**, **f**), MD-PRS (**g**, **h**), SCZ-PRS (**i**, **j**). Mental disorder genome-wide summary statistics (ADHD-PGC/iPSYCH, ASD-PGC/iPSYCH, BP-PGC, MD-PGC/UKBB, and SCZ-PGC) were used to construct Z-standardised PRS(C + T) in TEDS (ADHD-PRS, ASD-PRS, BP-PRS, MD-PRS, and SCZ-PRS) at multiple *P*-value thresholds. Association analyses with social behaviour (low-prosociality and peer-problem scores) were conducted using negative binomial regression (non-adjusted for cross-disorder PRS effects; multiple-testing corrected one-sided *P*-value: *P ≤ 0.0017). ADHD Attention-deficit/hyperactivity disorder, ASD Autism spectrum disorders, BP Bipolar disorder, C + T clumping and thresholding, iPSYCH Lundbeck Foundation Initiative for Integrative Psychiatric Research, MD Major depression, PGC Psychiatric Genomics consortium, PRS Polygenic risk scores, *P*_T_ PRS *P*-value threshold, SCZ Schizophrenia, TEDS Twins Early Development Study. Low-prosociality and peer-problem scores were assessed using the Strengths-and-Difficulties questionnaire.

Follow-up analyses in TEDS: Subsequently, we studied the univariate association of PRS(C + T) for ADHD, ASD, BP, MD, and schizophrenia risk (at 0.001 ≤ *P*_T_ < 1) with 15 ALSPAC-matching population-based social-behavioural measures in TEDS. Parent- and teacher-reported low-prosociality and peer-problem scores were longitudinally assessed between 4 and 16 years, showing skewed distributions (Table 1; Supplementary Table 11). At *P*_T_ ≤ 0.1, we replicated evidence for association between social-behavioural scores and polygenic risk for ADHD, MD and schizophrenia (Fig. 2; Supplementary Tables 12, 13; multiple-testing threshold: *P* ≤ 0.0017). In addition, we observed evidence for association between ASD-PRS and peer problems that was strongest for parent-reported scores at 11 years (β_ASD_11Y_(SE) = 0.093(0.018), ΔMcFadden’s-R^2^ = 0.0015, *P* = 2.7 × 10^–7^; Fig. 2c, d). There was no association between BP-PRS and any studied social trait (Fig. 2e, f).

### Stage 2: Meta-regression of polygenic association signals in ALSPAC and TEDS

For each disorder, we combined univariate polygenic PRS(C + T) estimates at *P*_T_ ≤ 0.1 for 29 SDQ-based social scores from both ALSPAC and TEDS, using a mixed-effects meta-regression approach (multiple-testing threshold: *P* ≤ 0.01). Specifically, we modelled heterogeneity in PRS effects as predicted by age-, reporter-, and trait-specific differences in social behaviour, captured by fixed-effect meta-regression estimates *θ*. For each disorder, we first fitted a full meta-regression model and, subsequently, dropped predictors to identify the most parsimonious model based on likelihood-ratio tests (Supplementary Tables 14, 15; Supplementary Figs. 4–7).

Meta-regression analyses revealed evidence for association between social behaviour and PRS for ADHD, ASD, MD, and schizophrenia, but not BP. Across disorders, polygenic effects varied with age, reporter, and, especially, social trait (Table 2). As there was little evidence for cohort-specific fixed effects (Supplementary Tables 14, 15), these effects were omitted from the most parsimonious models throughout. For ADHD-PRS, the most parsimonious meta-regression model provided evidence for an increase in PRS effects with age (*θ*_age(Y)_(SE) = 0.0025(8.9 × 10^−4^), *P* = 0.0042), teacher-reported scores (*θ*_teacher_report_(SE) = 0.044(0.0085), *P* = 2.5 × 10^−7^) and peer problems (*θ*_peer_problems_(SE) = 0.03(0.0089), *P* = 7.3 × 10^−4^). Likewise, the meta-regression model for MD-PRS showed an increase in PRS effect with age (*θ*_age(Y)_(SE) = 0.0035(9.5 × 10^−4^), *P* = 1.9 × 10^−4^) and peer problems (*θ*_peer_problems_(SE) = 0.048(0.0093), *P* = 2.8 × 10^−7^). In contrast to ADHD and MD, the most parsimonious model for schizophrenia revealed a decrease in PRS effects for peer problems (*θ*_peer_problems_(SE) = −0.027(0.0094), *P* = 0.0033). As there was a trend for a small positive age-effect that captured a considerable proportion of effect heterogeneity (ΔR^2^_Age_ = 0.33), this effect was retained in the model. For ASD-PRS, we observed an increase in PRS effect for peer problems (*θ*_peer_problems_(SE) = 0.037(0.0083), *P* = 7.9 × 10^−6^). The most parsimonious model for BP-PRS revealed little evidence for association with any social symptoms.

**Table 2:** Mixed-effects meta-regression of mental disorder PRS effects on social-behavioural symptoms.

		Non-adjusted for cross-disorder effects				Adjusted for cross-disorder effects			
	Parameter		*θ* (SE)	*Z*-value	*P*-value		*θ* (SE)	*Z*-value	*P-*value
ADHD-PRS		*R*^*2*^ = 0.88				*R*^*2*^ = 0.83			
	Intercept (Age 4, parent-reported, low prosociality)		−0.015 (0.010)	−1.49	0.14		−0.017 (0.011)	−1.50	0.13
	Age (Centred at 4 years)		0.0025 (0.00089)	2.86	0.0042		0.002 (0.001)	1.96	0.05
	Reporter (Teacher-reported)		0.044 (0.0085)	5.16	2.5 × 10^−7^		0.046 (0.0083)	5.56	2.6 × 10^−8^
	Trait (Peer problems)		0.03 (0.0089)	3.38	7.3 × 10^−4^		0.03 (0.0058)	5.20	2.0×10^−7^
ASD-PRS		*R*^*2*^ = 0.58				*R*^*2*^ = 0.67			
	Intercept (Low prosociality)		0.021 (0.0063)	3.36	7.7 × 10^−4^		0.017 (0.0049)	3.49	4.8 × 10^−4^
	Trait (Peer problems)		0.037 (0.0083)	4.47	7.9 × 10^−6^		0.025 (0.0054)	4.51	6.3 × 10^−6^
BP-PRS		*R*^*2*^ = 0.00				*R*^*2*^ = 0.00			
	Intercept		0.0054 (0.0056)	0.98	0.33		0.00056 (0.0054)	0.10	0.92
MD-PRS		*R*^*2*^ = 0.84				*R*^*2*^ = 0.81			
	Intercept (Age 4, low prosociality)		−0.018 (0.011)	−1.66	0.096		−0.021 (0.012)	−1.68	0.09
	Age (Centred at 4 years)		0.0035 (0.00095)	3.74	1.9 × 10^−4^		0.0027 (0.0011)	2.38	0.02
	Trait (Peer problems)		0.048 (0.0093)	5.14	2.8 × 10^−7^		0.051 (0.0068)	7.46	8.8 × 10^−14^
Schizophrenia-PRS		*R*^*2*^ = 0.45				*R*^*2*^ = 0.70			
	Intercept (Age 4, low prosociality)		0.017 (0.011)	1.55	0.12		0.026 (0.014)	1.91	0.06
	Age (Centred at 4 years)		0.0018 (0.00096)	1.86	0.063		0.0013 (0.0013)	0.99	0.32
	Trait (Peer problems)		−0.027 (0.0094)	−2.94	0.0033		−0.043 (0.0076)	−5.72	1.1 × 10^−8^

PRS(C + T) association effects for ADHD, ASD, BP, MD and schizophrenia risk on social behaviour (negative binominal model) were combined across 29 social symptoms (14 ALSPAC-based + 15 TEDS-based; at *P*_T_ ≤ 0.1) for each disorder using mixed-effects meta-regressions, accounting for phenotypic correlations between social scores. Here, the most parsimonious models are shown with fixed effect predictors (*θ*) of PRS effect heterogeneity including age- (centred at 4 years), reporter- (parent versus teacher reports), and trait-specific differences in social behaviour (low prosociality versus peer problems). The most parsimonious model for each disorder was identified using meta-regression combining univariate PRS effects that were non-adjusted for cross-disorder PRS effects. For comparison, corresponding estimates are given here for meta-regressions combining univariate PRS effects that were adjusted for cross-disorder PRS effects. R^2^ is defined as the ratio of explained variance to total variance in univariate PRS effects β.

*ADHD* Attention-Deficit/Hyperactivity Disorder, *ALSPAC* Avon Longitudinal study of Parents and Children, *ASD* Autism spectrum disorder, *BP* Bipolar disorder, *C* *+* *T* clumping and thresholding, *MD* Major depression, *PRS* Polygenic risk scores, *P*_*T*_ PRS threshold, *TEDS* Twins Early Development Study.

Multiple-testing corrected *P*-value: *P* ≤ 0.01.

**Fig. 3:**
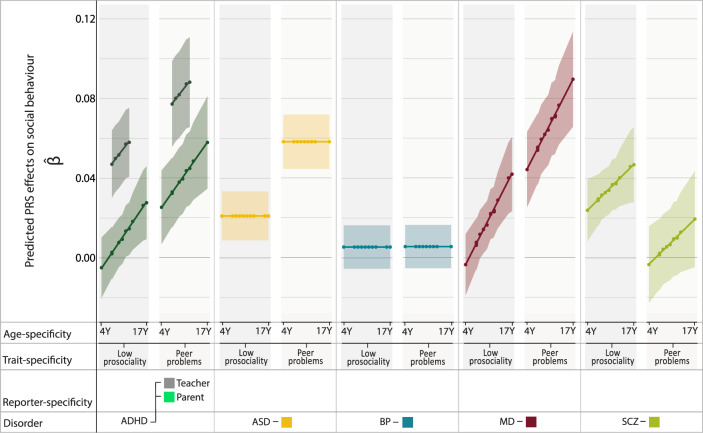
Meta-analytically predicted PRS effects for mental disorder with social behaviour. For each disorder (ADHD, ASD, BP, MD and schizophrenia) 29 SDQ-based PRS(C + T) effects (negative binominal model non-adjusted for cross-disorder PRS effects) from ALSPAC and TEDS (at *P*_T_ ≤ 0.1) were combined using mixed-effects meta-regression and predicted by age-, reporter- (parent versus teacher), and trait- (low prosociality versus peer problems) specific social symptoms. Based on the most parsimonious model, predicted PRS effects on social behaviour ($\hat \beta$) are shown as meta-regression lines with dots corresponding to the predicted input values and the shaded area corresponding to 95%-confidence intervals. ADHD Attention-Deficit/Hyperactivity Disorder, ALSPAC Avon Longitudinal study of Parents and Children, ASD Autism spectrum disorders, BP Bipolar disorder, C + T clumping and thresholding, MD Major depression, PRS Polygenic risk scores, *P*_T_ PRS threshold, SCZ Schizophrenia, SDQ Strengths-and-Difficulties questionnaire, TEDS Twins Early Development Study, Y years.

Predicted heterogeneity in PRS effects for ADHD, ASD, MD and schizophrenia (Fig. 3; Supplementary Figs. 4–7) can be summarised as follows: Meta-analytically predicted PRS effects ($\hat \beta$) indicated an association of ADHD-PRS with low prosociality based on teacher-reports ($\hat \beta$_ADHD_7Y_(SE) = 0.047(0.0086) to $\hat \beta$_ADHD_12Y_(SE) = 0.058(0.0088)) and for parent-reports only from 11 years onwards ($\hat \beta$_ADHD_11Y_(SE) = 0.013(0.0066) to $\hat \beta$_ADHD_17Y_(SE) = 0.028(0.0093)), but not between 4 to 10 years ($\hat \beta$_ADHD_4Y_(SE) = −0.0049(0.0077) to $\hat \beta$_ADHD_10Y_(SE) = 0.0094(0.0063)); ADHD-PRS were also associated with peer problems based on both parent-reports ($\hat \beta$_ADHD_4Y_(SE) = 0.025(0.0094) to $\hat \beta$_ADHD_17Y_(SE) = 0.058(0.012)) and teacher-reports ($\hat \beta$_ADHD_7Y_(SE) = 0.077(0.011) to $\hat \beta$_ADHD_12Y_ = 0.088(0.012)). Polygenic association with MD-PRS increased with age and was larger for peer problems ($\hat \beta$_MD_4Y_(SE) = 0.044(0.0097) to $\hat \beta$_MD_17Y_(SE) = 0.091(0.012)) than low prosociality ($\hat \beta$_MD_4Y_(SE) = −0.0033(0.0078) to $\hat \beta$_MD_17Y_(SE) = 0.042(0.0095)) with evidence for an association with low prosociality only from 9 years onwards ($\hat \beta$_MD_9Y_(SE) = 0.014(0.0061)). In contrast, association effects of schizophrenia-PRS risk with social behaviour were only found for low prosociality ($\hat \beta$_SCZ_4Y_(SE) = 0.024(0.0079) to $\hat \beta$_SCZ_17Y_(SE) = 0.047(0.0096)), but not peer problems ($\hat \beta$_SCZ_4Y_(SE) = −0.0036(0.0098) to $\hat \beta$_SCZ_17Y_(SE) = 0.019(0.0120)). ASD-PRS association effects were stable across age, but larger for peer problems ($\hat \beta$_ASD_(SE) = 0.058(0.0070)) than low prosociality ($\hat \beta$_ASD_(SE) = 0.021(0.0063)).

Analogous meta-regression analyses, combining univariate PRS-CS effects showed consistent results in ALSPAC, highlighting the robustness of our findings (Supplementary Table 16).

Adjusting univariate mental disorder PRS(C + T) effects for each other (cross-disorder adjusted PRS effects) in ALSPAC (Supplementary Tables 17, 18) and TEDS (Supplementary Tables 19, 20) strengthened, when meta-analysed, the evidence for reporter- (ADHD only) and trait-specific heterogeneity in ADHD, ASD, MD and schizophrenia PRS effects. In contrast, age-specific heterogeneity in PRS effects, if present, was either attenuated or abolished (Table 2, Supplementary Tables 21, 22). Together, these findings demonstrate distinct reporter- and trait-specific association profiles for social behaviour across mental health conditions, but shared genetic liability for age-related profiles.

### Biological-pathway-based PRS analyses

PRS(C + T) approaches can be extended to explore biological processes (PRSet) [38]. Conditional on marker sets selected at *P*_T_ ≤ 0.1 (baseline), we screened for each disorder whether pathway-based PRS (7,481 GOBP gene sets) can re-capture genetic links with low-prosociality and peer-problem scores at the same or higher strength, as observed at baseline (Supplementary Tables 23–27). For simplicity, we focussed on the four most strongly associated social behaviours, as predicted by meta-regression (i.e. low-prosociality and peer-problem scores for teacher reports at 11 years and parent reports at 17 years). Pathway-based ADHD-PRS for sensory perception of sour taste captured the association with teacher-reported peer problems at 11 years in strength and, approximately, in magnitude (sensory perception: *β*(SE) = 0.11(0.024), *P* = 1.1 × 10^−5^; baseline: *β*(SE) = 0.11(0.025), *P* = 1.2 × 10^−5^). Pathway-based schizophrenia-PRS for telencephalon regionalisation reflected the association with parent-reported low prosociality at 17 years (telencephalon regionalisation: *β*(SE) = 0.064(0.015), *P* = 2.8 × 10^−5^; baseline: *β*(SE) = 0.064(0.015), *P* = 2.8 × 10^−5^). Similarly, pathway-based schizophrenia-PRS for macrophage differentiation and protein polyubiquitination retained the association with teacher-reported low prosociality at 11 years (e.g. macrophage differentiation: β(SE) = 0.087(0.019), *P* = 3.6 × 10^−6^; protein polyubiquitination: *β*(SE) = 0.074(0.019), *P* = 7.9 × 10^−5^; baseline: *β*(SE) = 0.072(0.019), *P* = 1.5 × 10^−4^). For all other disorders, either the baseline-PRS did not pass the multiple-testing threshold, or no pathway-PRS reached the strength of the baseline-PRS effect.

## Discussion

Investigating polygenic links between risk for mental disorder and population-based social behaviour, this study identified differences in genetic associations across a spectrum of social-behavioural difficulties. We observed robust evidence for shared genetic influences between child and adolescent social difficulties and polygenic risk for ADHD, MD and schizophrenia across two large UK population-based cohorts conducting a univariate association approach. Combining univariate findings in a meta-regression framework, we identified further evidence for association between ASD risk and social difficulties. Here, we show that the identified meta-analytic association profiles systematically vary with age-, reporter- and trait-specific social symptoms across disorders. These findings suggest a diverse genetic landscape of social phenotypes that is differentially shared with risk for mental disorder. As such, our results refine previous research demonstrating the genetic overlap of psychiatric risk with social phenotypes, such as reported for emotion recognition in childhood and adolescence [14, 18], self-reported empathy [15], loneliness [39], and sociability [17] in adults.

Age-specific increases in polygenic overlap with social behaviour from 4 years onwards were shared across ADHD, MD and schizophrenia risk, as demonstrated by cross-disorder adjusted analyses. These findings confirm previously reported developmental changes in the genetic overlap of schizophrenia risk with social communication [16]. A developmental increase in genetic association effects is also in line with the typical onset of MD and schizophrenia during adolescence and adult life [40, 41]. Genetic associations at earlier ages may link to subthreshold social difficulties preceding clinical diagnosis [42, 43] or early-onset cases, which are thought to convey more severe symptoms [44, 45]. For ADHD, a typical childhood-onset disorder, the age-specific increase in association may imply that genetic links progress into adulthood [46].

Conversely, the lack of age-specific changes in the association of ASD risk with social behaviour suggests, given sufficient power assuming fixed trait-disorder covariance, that these polygenic links may involve social problems that already emerge before or at the age of 4 years and remain developmentally stable, consistent with early social core deficits in ASD [2]. These findings contrast the developmental decline in the genetic overlap of ASD risk with social communication scores that was previously reported [16], possibly reflecting differences in social behaviour versus social-communication-related skills where the latter rely more strongly on social cognition and verbal and non-verbal communication [8].

For ADHD risk only, we identified distinct reporter-specific heterogeneity in PRS effects, with stronger genetic links for teacher- compared to parent-reported social symptoms, irrespective of cross-disorder adjustment. School environments may, specifically, expose behavioural difficulties of children with ADHD. Social behaviour at school, as reported by teachers, evaluates rule-oriented behaviour [47], but also adequate peer-peer interactions among children of the same age. Problems may arise due to children’s high levels of distractibility but also their disruptive/oppositional behaviours [1].

For ADHD, ASD, MD and schizophrenia risk, we found evidence for distinct social trait-specific heterogeneity in PRS effects that was robust to cross-disorder adjustment. The most pronounced differences in association patterns were identified for schizophrenia compared to ADHD, ASD, and MD risk, as captured by the opposite direction of the meta-regression effect theta. Schizophrenia risk was exclusively associated with low prosociality, but not peer problems, possibly reflecting specific impairments in social cognition and a lack of social interest and empathy in psychotic disorders [6]. In contrast, there was a stronger genetic association of ADHD, ASD, and MD risk with peer problems, compared to prosocial scores. Despite a similarity in effect direction, these associations showed disorder-specific effect variation, consistent with socially disruptive behaviour and poor social skills in ADHD, ASD and MD, contributing to difficulties in communication, emotion regulation, executive functioning, and/or social isolation [5, 48, 49].

Together, our findings demonstrate shared genetic liabilities across mental disorders describing age-related changes in genetic overlap with social behaviour, strengthening the hypothesis of a neurodevelopmental continuum [50] and the need for a developmental perspective in clinical practice [51]. In contrast, the robust reporter- and trait-specific heterogeneity in polygenic associations with social behaviour suggests that genetic risk across mental health conditions, as studied here, is also multidimensional [52, 53]. Disorder-specific association profiles may, therefore, help refining diagnostic criteria and targeted treatment strategies, especially, for psychotic versus non-psychotic disorders [54]. Similarities in profiles may still exist among highly-correlated mental conditions (not investigated here), as for example between MD and anxiety disorder (r_g_ > 0.8) [55].

For ADHD and schizophrenia risk, pathway-PRS analyses in ALSPAC identified candidate molecular mechanisms underlying the overlap with social behaviour. The association between ADHD-PRS and teacher-reported peer problems was most strongly linked to sensory processing of sour taste, in line with frequently altered sensory processing skills in children with ADHD [56]. The association between schizophrenia-PRS and parent-reported low prosociality at 17 years could be recaptured by pathway-PRS for telencephalon regionalisation, a biological process that is implicated in the aetiology of schizophrenia [57]. The association between schizophrenia-PRS and teacher-reported low prosociality at 11 years was related to processes of macrophage differentiation and protein polyubiquitination. Increased macrophages have been linked to reduced adult neurogenesis in the subventricluar zone in schizophrenia [58], and protein polyubiquitination has been described as a molecular marker for schizophrenia [59]. There was little support for other pathway PRS recapturing polygenic association at the same strength and magnitude as observed at baseline (*P*_T_ ≤ 0.1). However, substantial permutation and replication analyses, beyond the scope of this work, will be required to refine the insight into the aetiological mechanisms underlying the observed polygenic associations.

Finally, the absence of genetic interrelationships with BP is consistent with previous reports studying social problems in childhood [60] and self-reported empathy [15] and sociability [17] in adulthood. This lack of association may either reflect lack of power, which is unlikely given power estimations of >80% when assuming fixed trait-disorder covariance (Supplementary Fig. 2), or suggests that social symptoms during childhood and adolescence may not be directly involved in the genetic aetiology of BP.

Our study has several strengths and limitations: We investigated polygenic links of multiple mental health conditions with two social traits as reported by parents and teachers across 13 years of child and adolescent development. Adopting a two-stage research design, we first studied genetic association in two large UK population-based cohorts using a count data approach and then modelled heterogeneity in polygenic estimates with mixed-effects meta-regressions. Our study had sufficient power to detect polygenic association with both parent- and teacher-reported social behaviour across all studied disorder PRS (Supplementary Fig. 2), despite known bias affecting parent-reported measures [21]. However, consistent with other PRS analyses [16], effect sizes were small with little predictive ability at the individual level. Also, due to different sets of risk-increasing alleles analysed, a direct comparison of PRS effect size across disorders is not meaningful here, although we studied cross-disorder adjusted PRS estimates. In addition, we exclusively investigated social symptoms with the SDQ, a widely used instrument to screen for mental disorders [61]. Different instruments, including those assessing reciprocal social interactions, might capture a wider symptom spectrum, in particular for ASD. Furthermore, polygenic signals might be biased by population-based phenomena such as dynastic effects and non-random mating [62], but also non-random missingness [63, 64]. However, such bias would uniformly affect all ascertained SDQ scores, resulting in homogeneous and not heterogeneous genetic association profiles. Nonetheless, it is possible that the age-specific increase in genetic overlap with disorder has been underestimated, as study participation has been linked to lower PRS for psychopathology [64]. Finally, population-based cohorts with a similar size such as ALSPAC and TEDS can predominantly detect the genetic contribution of common and low-frequent but not rare variation [65]. Further studies should refine our findings by replicating analyses across a wider spectrum of social phenotypes in European and non-European cohorts to promote the translation into precision medicine [54].

In conclusion, our findings reveal differences in the social genetic architecture across mental disorders when studying polygenic associations with population-based social behaviour. Age-related variation in polygenic overlap with social behaviour was shared across mental health conditions, while reporter- and social trait-specific variation captured disorder-specific profiles. Together, our findings demonstrate that social symptoms represent a heterogeneous spectrum of related endophenotypes.

## Supplementary information

Supplementary information

Supplementary tables
