# Supplementary material for: Polygenic risk for mental disorder reveals distinct association profiles across social behaviour in the general population
Source: Mol Psychiatry. 2022 Feb 28;27(3):1588–98. doi: 10.1038/s41380-021-01419-0 (PMC9095485; doi:10.1038/s41380-021-01419-0)
Supplement: Supplementary file 1 — Supplementary information [file 41380_2021_1419_MOESM1_ESM.docx]

**Supplementary information**

Polygenic risk for mental disorder reveals distinct association profiles across social behaviour in the general population

[**Supplementary Methods** 2](#_Toc83369442)

[*Genome-wide summary statistics of mental disorders* 2](#_Toc83369443)

[*Description of ALSPAC* 4](#_Toc83369444)

[*Social behavioural measures in the general population* 4](#_Toc83369445)

[*Genome-wide genotyping information in ALSPAC and TEDS* 5](#_Toc83369446)

[*SNP-h^2^ analyses using genome-wide summary statistics* 5](#_Toc83369447)

[*Univariate polygenic scoring analyses using clumping and thresholding: PRS(C+T)* 7](#_Toc83369448)

[*Univariate PRS analyses in ALSPAC using PRS-CS* 8](#_Toc83369449)

[*Power of univariate PRS(C+T) analyses in ALSPAC* 9](#_Toc83369450)

[*Mixed-effects meta-regression* 10](#_Toc83369451)

[*Gene ontology biological pathway-based PRS analyses in ALSPAC* 11](#_Toc83369452)

[*Web resources* 12](#_Toc83369453)

[**Supplementary Figures** 13](#_Toc83369454)

[**Supplementary References** 21](#_Toc83369455)

# **Supplementary Methods**

## *Genome-wide summary statistics of mental disorders*

ADHD-PCG/iPSYCH: We used GWAS summary statistics for clinical ADHD from a meta-analysis combining samples of the Psychiatric Genomic Consortium (PGC) and the Danish Lundbeck Foundation Initiative for Integrative Psychiatric Research (iPSYCH), comprising in total 20,183 ADHD cases and 35,191 controls^1^ (Supplementary Table 1). ADHD-iPSYCH is a case-control study (21.6% female ADHD-cases, and ~49% female controls) embedded within a nationwide population-based sample^1^. All participants were of European ancestry with an age range spanning infancy to adulthood^2^. ADHD cases (N=14,584) were identified via the Danish Psychiatric Central Research Register based on diagnoses by psychiatrists at a psychiatric hospital according to the International Classification of Diseases (ICD10, F90.0)^3^. Controls (N=22,492) were randomly selected from the same nationwide birth cohort and did not have a diagnosis of ADHD (F90.0) or moderate-severe intellectual disability (F71-F79)^1,2^. iPSYCH samples were derived from the Danish Newborn Screening Biobank hosted by Statens Serum Institute. Genotypes were determined using the Illumina’s Beadarrays (PsychChip; Illumina, CA, San Diego, USA). Note that ADHD-iPSYCH controls are shared with the ASD-iPSYCH sample. ADHD-PGC comprises seven case-control samples, and four family-based samples of predominantly white European ancestry including a total of 4,163 cases (~12-54% females) and 12,040 controls/pseudo-controls (~12-61% females), with ages spanning childhood to adulthood from age 3 onwards. Diagnoses of ADHD were based on the Diagnostic and Statistical Manual of Mental Disorders (DSM-III, DSM-IV, DSM-TR) or the ICD-10. Note, that ADHD-cases might have an additional diagnosis of ASD.

ASD-PGC/iPSYCH: GWAS summary statistics for clinical ASD were obtained from a meta-analysis combining samples from PGC and iPSYCH, with a total of 18,381 ASD cases and 27,969 controls^4^ (Supplementary Table 1). ASD-iPSYCH includes 13,076 cases and 22,664 controls, and is also based on the nationwide population-based iPSYCH sample. ASD cases (19-25.3% females) were identified via the Danish Psychiatric Central Research Register. ASD diagnoses were made by a psychiatrist in 2013 or earlier according to ICD-10 diagnoses of childhood autism, atypical autism, Asperger’s syndrome, other pervasive developmental disorders, and pervasive developmental disorder, unspecified (F84.0, F84.1, F84.5, F84.8, F84.9), with ages of first diagnoses ranging from 5 to 15 years. Controls (49.3% females) were randomly selected and did not have a diagnosis of ASD by 2013. ASD-PGC comprises five family-based cohorts of predominantly European ancestry^4,5^. ASD cases (N= 5,305) in PGC were identified based on research standard tools and expert clinical consensus diagnoses^4^. Individuals were excluded if assessments took place before the age of 36 months or if diagnostic criteria did not meet criteria of Autism Diagnostic Interview-Revised (ADI-R) or the Autism Diagnostic Observation Schedule (ADOS) domain scores^5^. Information on the male-female ratio was not available^6^.

BP-PGC: BP-PGC summary statistics are based on a meta-analysis of 32 cohorts with a total of 20,352 cases and 31,358 controls^7^ (Supplementary Table 1), all of European descent and aged 17 years or older^7^. There was no information on the male-female ratio^7^. BP diagnoses were made using structured diagnostic instruments from assessments by trained interviewers, clinician-administered checklists or medical record reviews, and were based on international consensus criteria (DSM-IV or ICD-10) for a lifetime diagnosis of BP^7^. In most cohorts, controls were screened for the absence of lifetime psychiatric disorders and randomly selected from the population^7^.

MD-PGC/UKBB: MD summary statistics are based on a meta-analysis of PGC and UK Biobank (UKBB) samples, with a total of 170,756 cases and 329,443 controls^8^ (Supplementary Table 1). MD-PGC includes 33 European ancestry cohorts with a total of 43,204 cases and 95,680 controls^9^. In most cohorts, cases were selected based on DSM-IV, ICD-10, or ICD-9, and did not have a diagnosis of BP, non-affective psychosis, MD related to substance use disorder, mania or hypomania. Controls were predominantly selected using exclusion criteria with respect to a diagnosis of MD or depressive symptoms, BP or any other severe (mental) illnesses^9^. Information on participant age and a male-female ratio was not available^9,10^. The MD-UKBB sample is a UK population-based study recruited from the UK Biobank^8^ including 127,552 cases (65% females), and 233,763 controls (48% females) with an age range spanning from 39 to 73 years^10^. Cases were selected according to three depression phenotypes including broad depression (self-reported help-seeking behaviour for mental health difficulties from either a general practitioner or psychiatrist), probable MD^11^, and ICD-coded MD based on a primary or secondary diagnosis of MD in hospital admission records (ICD-9/10 codes F32, F33, F34, F38, F39 or non-cancer illness code 1286)^10^. Cases were excluded from the analysis if they were identified with bipolar disorder, schizophrenia, or personality disorder, and if there was evidence for being prescribed any antipsychotic medication^10^. Exclusion criteria from the control sample were a diagnosis of a depressive mood disorder from hospital admission records (ICD-9/10 codes F32, F33, F34, F38, and F39), a reported prescription for antidepressants or self-reported depression^10^ (Supplementary Table 1).

SCZ-PGC: Summary statistics are based on a meta-analysis of 46 ancestry-matched non-overlapping case-control samples and 3 family-based samples of European ancestry including a total of 33,640 cases and 43,456 controls/pseudo-controls^12^. Individuals with schizophrenia or schizoaffective disorder were included as cases, based on research-based assessment criteria or diagnoses by a clinician. A detailed cohort specific ratio of males is described elsewhere showing a balanced male/female representation in the majority of the cohorts^12^. Information on the age representation was not consistently available^12^ (if reported, >18 years of age, Supplementary Table 1).

In PGC and iPSYCH based studies, quality control, imputation, and association analyses were carried out for each cohort individually according to standard PGC settings using the PGC “ricopili” pipeline^12^. Genotypes were imputed against 1000 Genomes Project reference panels^13,14^ (Supplementary Table 1). Quality control criteria for the MD-UKBB sample are described in detail by Howard and colleagues^10^, and genotypes were imputed with IMPUTE4 against the HRC reference template^15,16^ (Supplementary Table 1).

## *Description of ALSPAC*

Pregnant women resident in Avon, UK with expected dates of delivery 1st April 1991 to 31st December 1992 were invited to take part in the study^17,18^. The initial number of pregnancies enrolled is 14,541 (for these at least one questionnaire has been returned or a “Children in Focus” clinic had been attended by 19/07/99). Of these initial pregnancies, there was a total of 14,676 foetuses, resulting in 14,062 live births and 13,988 children who were alive at 1 year of age. When the oldest children were approximately 7 years of age, an attempt was made to bolster the initial sample with eligible cases who had failed to join the study originally. As a result, when considering variables collected from the age of seven onwards (and potentially abstracted from obstetric notes) there are data available for more than the 14,541 pregnancies mentioned above. The number of new pregnancies not in the initial sample (known as Phase I enrolment) that are currently represented on the built files and reflecting enrolment status at the age of 24 is 913 (456, 262 and 195 recruited during Phases II, III and IV respectively), resulting in an additional 913 children being enrolled. The phases of enrolment are described in more detail in the cohort profile paper and its update. The total sample size for analyses using any data collected after the age of seven is therefore 15,454 pregnancies, resulting in 15,589 foetuses. Of these 14,901 were alive at 1 year of age. A 10% sample of the ALSPAC cohort, known as the Children in Focus (CiF) group, attended clinics at the University of Bristol at various time intervals between 4 to 61 months of age. The CiF group were chosen at random from the last 6 months of ALSPAC births (1,432 families attended at least one clinic). Excluded were those mothers who had moved out of the area or were lost to follow-up, and those partaking in another study of infant development in Avon. Please note that the study website contains details of all the data that are available through a fully searchable data dictionary and variable search tool.

## *Social behavioural measures in the general population*

The SDQ is a brief behavioural screening questionnaire capturing behavioural symptoms^19^. The SDQ prosocial behaviour subscale consists of the items: (1) "Considerate of other people's feelings"; (2) "Shares readily with other children"; (3) "Helpful if someone is hurt"; (4) "Kind to younger children"; (5) "Often volunteers to help others". Each item was scored as either "not true" (2), "somewhat true" (1) or "certainly true" (0), and eventually added to a total sum score. The total sum of prosocial behaviour item scores was reverse-coded, such that higher scores reflect lower prosociality. The SDQ peer problem subscale includes the items (1) "Rather solitary, tends to play alone"; (2) "Has at least one good friend"; (3) "Generally liked by other children"; (4)"Picked on or bullied by other children"; 5)"Gets on better with adults than with other children". Items (4) and (5) were reverse-coded such that the total sum score of all peer problem items indicates increased peer-related problems.

A description of study numbers and demographics for each assessed social score in ALSPAC and TEDS are detailed in Table 1.

## *Genome-wide genotyping information in ALSPAC and TEDS*

ALSPAC: Genome-wide genotyping in ALSPAC was performed using the Illumina HumanHap550 array^17,18^. Standard quality control was applied to the raw genome-wide data as described previously^20^. In brief, 8,981 subjects and 465,740 SNPs passed the quality control filters and were imputed to the Haplotype Reference Consortium (HRC) reference panel version r1.1 using the Sanger Imputation Server^15^. Only high-quality imputed SNPs (INFO > 0.8) with a genotype probability of > 0.8 in > 95% of the individuals, and a minor allele frequency (MAF) > 0.005 (*N*=7,337,399) were included in subsequent analyses.

TEDS: Genome-wide genotyping data were assessed using the Affymetrix GeneChip 6.0 or the Illumina HumanOmniExpressExome-8v1.2 arrays. Typical quality control procedures were followed and have been described in detail elsewhere^21^. In short, quality-controlled genotypes from the two platforms (7,289 individuals and 559,772 SNPs genotyped on Illumina and 3,057 individuals and 635,269 SNPs genotyped on Affymetrix) were imputed separately to the HRC reference panel version r1.1^15^, and then harmonised. Only high-quality imputed SNPs (INFO>0.75, genotyping-missingness<0.02, individual-missingness<0.02, MAF>0.005, and Hardy Weinberg equilibrium *P*-value>0.00001; *N*=7,363,646) were included for further analyses. The following exclusions were applied: extreme perinatal conditions and severe medical conditions.

## *SNP-h^2^ analyses using genome-wide summary statistics*

*Generation of ALSPAC summary statistics:* For SNP-heritability (SNP-h^2^) analyses of social behaviour in ALSPAC children, we conducted genome-wide association studies (GWAS) using untransformed social scores for both low prosociality (7, 10, 13, 14, and 17 years of age), and peer problems (7, 10, 13, 14, and 17 years of age). Avoiding data transformation, we use a count data-based regression approach to either regress positively skewed peer-problems or low-prosociality scores on allele dosage, age, sex, and the first two most significant ancestry-informative principal components (PC; correcting for subtle differences in population structure).

We apply a quasi-Poisson model that accommodates count data with a variance exceeding the mean (over-dispersion, Table 1).

The quasi-Poisson regression model was defined as

$\log(\mu)=\beta_{0}+ \beta_{{SNP}_{i}}x_{{SNP}_{ij}}+\beta_{age}x_{{age}_{j}}+\beta_{sex}x_{{sex}_{j}}+ \beta_{PC1}x_{{PC1}_{j}}+\beta_{PC2}x_{{PC2}_{j}}+\varepsilon_{j}$ [1]

$var\left( y \right)=\Phi\mu$ [2]

where log changes in $\mu$, the mean value of a social trait, are a function of $x_{{SNP}_{ij}}$ the allele dosage for the *i*-th high-quality imputed SNP for the *j-*th participant, $x_{{age}_{j}}$the age of the participant *j* at assessment, $x_{{sex}_{j}}$ the dummy coded sex of participant *j* with 1 indicating male and 0 female, and $x_{{PC1}_{j}}$ and $x_{{PC2}_{j}}$ the first two principal components for the *j-*th participant. The quasi-Poisson model accounts for over-dispersion in count data by defining the variance of y by the mean $\mu$ proportional to its dispersion parameter $\Phi$, where $\Phi$ is given by Pearson’s $X^{2}$ divided by the residual degrees of freedom (df): $\hat{\Phi}=\frac{X^{2}}{df}$ with $\hat{\Phi}$> 1 indicating over-dispersion^22^. Note, that a quasi-Poisson regression does not model an error distribution, with the deviance being identical to the one derived from a Poisson distribution. Therefore, the model fit cannot be assessed using a likelihood-ratio test^23^. However, compared to the conceptually related negative binomial model, the quasi-Poisson model is easier to integrate within a genome-wide analysis framework, due to less restrictive modelling assumptions^24^. We demonstrate an improved model fit using count data approaches, allowing for over-dispersion, by comparing the model fit of a negative binomial model with an ordinary least squares model (Supplementary Table 4). After creating genome-wide genotype dosage files in hdf5 file format, using the HASE framework^25^, we applied quasi-Poisson regressions using custom-based R scripts, as count models are not yet implemented in standard GWAS software.

*LDSC SNP-h^2^ analyses:* SNP-h^2^ for social behaviour (low-prosociality and peer-problem scores) and mental health conditions were estimated from GWAS summary statistics using unconstrained linkage disequilibrium score (LDSC) regression^26^ to inform power analyses.

SNP-h^2^ estimates describe here either the proportion of phenotypic variance (social behaviour) or liability to disorder, as tagged by common SNPs on genotyping arrays (Supplementary Tables 7 and 8). In LDSC, SNP-h^2^ estimates represent the slope of the regression of genome-wide Χ^2^-statistics against the corresponding LD scores, while the intercept minus one estimates the mean contribution of confounding bias due to the inflation in the mean Χ^2^-statistic^26^. For completeness, we also calculated genomic control λ (λ_GC_) estimates which assess the extent of inflation in GWAS test statistics due to population stratification^27^. SNP-h^2^ for mental disorders was estimated on the liability scale, assuming a population prevalence of 0.05 for ADHD^28^, 0.012 for ASD^4^, 0.006 for BP^29^, 0.162 for MD^30^, and 0.007 for schizophrenia^31^.

All analyses were performed with LDSC software using pre-computed LD scores based on European-ancestry samples of the 1000 Genomes European Project^26^.

## *Univariate polygenic scoring analyses using clumping and thresholding: PRS(C+T)*

Polygenic risk scores (PRS) for mental disorder were constructed for ALSPAC and TEDS participants using PLINK software^32^. Consistent with current guidelines^33^, clinical summary statistics from ADHD-PGC/iPSYCH, ASD-PGC/iPSYCH, BP-PGC, MD-PGC/UKBB, and schizophrenia-PGC were clumped (LD-r^2^>0.25, ±500 kb) using PLINK software^32^. Risk variants were selected from summary statistics across nine *P*-value thresholds (0.001≤*P*_T_<1). In ALSPAC, PRS were constructed for unrelated ALSPAC children and adolescents (genomic relatedness<0.125), based on high-quality imputed SNPs (INFO>0.8, 95%-posterior genotyping probability>0.9, MAF>0.005). In TEDS, PRS were generated for dizygotic twin pairs and a single twin from each monozygotic twin pair, using high-quality imputed SNPs in TEDS (INFO>0.75, genotyping-missingness<0.02, individual-missingness<0.02, MAF>0.005, and Hardy Weinberg equilibrium *P*-value>0.00001). The log odds of genetic SNP effects were aligned to indicate alleles with increased risk for mental disorder, and PRS were Z-standardised.

To estimate the association between polygenic risk for mental disorder and social behaviour in ALSPAC we applied a negative binomial regression where log changes in social behaviour are predicted by

$\log\left( {social score}_{j} \right)=\beta_{0}+ \beta_{PRS}x_{{PRS}_{j}}+\beta_{age}x_{{age}_{j}}+\beta_{sex}x_{{sex}_{j}}+ \beta_{PC1}x_{{PC1}_{j}}+\beta_{PC2}x_{{PC2}_{j}}$ [3]

with $x_{{PRS}_{j}}$being the polygenic risk score for participant *j*, $x_{{age}_{j}}$ the age of assessment for participant *j*, $x_{{sex}_{j}}$ the dummy coded sex for participant *j* with 1 indicating male and 0 female, and $x_{{PC1}_{j}}$ and $x_{{PC2}_{j}}$ the first two principal components (PC) for the *j*-th participant. The variance of negative binomial distribution is a function of the mean adjusted by the inverse of the overdispersion dispersion parameter $\Phi$, scaled by the square of the mean. For a count random variable (here social score), this is given as

$var\left( social scores \right)=\mu+\mu^{2}/\Phi$ [4]

To estimate cross-disorder adjusted PRS effects, disorder PRS effects were, in addition, adjusted for each other: [5]

$$\log\left( {social score}_{j} \right)=\beta_{0}+ \beta_{PRS_{ADHD}}x_{{PRS_{ADHD}}_{j}}+ \beta_{PRS_{ASD}}x_{{PRS_{ASD}}_{j}}+ \beta_{PRS_{BP}}x_{{PRS_{BP}}_{j}}+$$

$$\beta_{PRS\_MD}x_{{PRS\_MD}_{j}}+ \beta_{PRS\_SCZ}x_{{PRS\_SCZ}_{j}}+\beta_{age}x_{{age}_{j}}+\beta_{sex}x_{{sex}_{j}}+ \beta_{PC1}x_{{PC1}_{j}}+\beta_{PC2}x_{{PC2}_{j}}$$

The negative binomial model accounts for an over-dispersed error distribution in count data. The model can be fitted using a maximum likelihood approach with derived parameters following a defined probability distribution^23^. Thus, models can be compared using a likelihood-ratio test and the strength of the association can be assessed using quasi-R^2^ measures.

For analyses in TEDS, we implemented a generalized linear modelling approach, fitting a mixed-effects negative binomial model. This model, additionally, allows for a random intercept $\gamma_{j}$accounting for within family relatedness across each of the two dizygotic twins (twin 1 and 2) in family *j* with $\gamma_{j}$ ~N(0,$\sigma_{\gamma}^{2}$) capturing the change in the intercept for both twins in family *j*:

$\log\left( {social score}_{tj} \right)=\beta_{0}+ \beta_{PRS}x_{{PRS}_{tj}}+\beta_{age}x_{{age}_{tj}}+\beta_{sex}x_{{sex}_{tj}}+\beta_{covar}x_{{covar}_{tj}}+\gamma_{j}$ [6]

TEDS-specific covariates (covar) include the first ten PCs, genotyping-batch and genotyping-chip effects.

Additionally, we estimated cross-disorder adjusted PRS effects in TEDS implementing the following multiple regression form of the model: [7]

$$\log\left( {social score}_{tj} \right)=\beta_{0}+ \beta_{PRS_{ADHD}}x_{{PRS_{ADHD}}_{tj}}+ \beta_{PRS_{ASD}}x_{{PRS_{ASD}}_{tj}}+$$

$$\beta_{PRS\_BP}x_{{PRS\_BP}_{tj}}+ \beta_{PRS\_MD}x_{{PRS\_MD}_{tj}}+ \beta_{PRS\_SCZ}x_{{PRS\_SCZ}_{jt}}+\beta_{age}x_{{age}_{jt}}+\beta_{sex}x_{{sex}_{tj}}+\beta_{covar}x_{{covar}_{tj}}+\gamma_{j}$$

To estimate the improvement in model fit attributable to $\beta_{PRS}$ as a predictor, we assessed ΔMc Fadden’s R^2^ which was given by

$\Delta{Mc Fadden^{'}s R}^{2}=\left( 1- \frac{LL\left( Full model \right)}{LL\left( Intercept model \right)} \right)- \left( 1- \frac{LL\left( Baseline model \right)}{LL\left( Intercept model \right)} \right)$ [8]

as the difference between one minus the proportion of the log likelihood (LL) of the full model representing the regression function including $\beta_{PRS}$ as a predictor against the LL of the intercept model, and one minus the proportion of the LL of the baseline model representing the regression function excluding $\beta_{PRS}$ as a predictor against the LL of the intercept model. For the estimation of ΔMc Fadden’s R^2^ of cross-disorder adjusted PRS effects, the LL of the baseline models was based on regression equations that dropped the respective disorder PRS effect.

## *Univariate PRS analyses in ALSPAC using PRS-CS*

To assess the robustness of our findings across different PRS methods, we repeated PRS analyses in ALSPAC using PRS-CS^34^, a Bayesian-based approach that adjusts SNP effect sizes for LD by applying a continuous-shrinkage parameter. Here, we selected the auto-option for a fully Bayesian estimation of the shrinkage parameter phi. Furthermore, we applied the software’s default settings by setting parameter a in the gamma-gamma prior to 1, parameter b in the gamma-gamma prior to 0.5 and selecting 1,000 Markov Chain Monte Carlo iterations, 500 burn-in iterations, and a Markov chain thinning factor of 5. As LD reference file, we used the UK Biobank European reference panel recommended on the software’s git-hub page. As described for the PRS(C+T) approach above, PRS were constructed for unrelated ALSPAC children and adolescents (genomic relatedness<0.125), based on high-quality imputed SNPs (INFO>0.8, 95%-posterior genotyping probability>0.9, MAF>0.005). Once per-allele posterior SNP effect sizes were calculated in PRS-CS, PRS scores were calculated in PLINK^32^ and z-standardised. As described for the PRS(C+T) approach above, we fitted a negative binomial regression model to the data regressing social-behavioural traits on the respective disorder-based PRS score, accounting for sex, age and the first two ancestry-informative principal components, and, subsequently, assessed ΔMc Fadden’s R^2^.

## *Power of univariate PRS(C+T) analyses in ALSPAC*

We assessed the power to detect univariate polygenic trait-disorder associations in ALSPAC with PRS(C+T) using the R software package avengeme^35^. Power estimates were informed by the estimated SNP-h^2^ of the disorder, based on summary statistics, the sample sizes of discovery and target samples, and the number of genetic variants used to construct PRS (Table 1, Supplementary Tables 1,7). We set the proportions of SNPs with no causal effect on the discovery sample (i.e. the presence of large single genetic effects, pi0) to pi0= 0.95. We estimated power for two covariance settings across a range of applied *P*-value selection thresholds (0.001≤*P*_T_<1): (1) a fixed trait-disorder covariance governed by the SNP-h^2^ of the disorder and (2) the estimated genetic trait-disorder covariance as observed in the univariate regression model. Note, that a drop in power of the latter approach compared to condition 1, reflects the lack of observed genetic covariance, and is, thus, not governed by a potential lack of SNP-h² of the discovery sample. We also report the estimated trait-disorder covariance for all studied mental disorder PRS across a range of different *P*-value thresholds (0.001≤*P*_T_<1).

## *Mixed-effects meta-regression*

In order to investigate variation in univariate PRS effects capturing the association between polygenic risk for mental disorder and social traits, we conducted mixed-effects meta-regressions (R:metafor^36^). Specifically, we combined for each disorder the 14 ALSPAC-based and 15 TEDS-based PRS effects for SDQ-based social scores (β_i_, *i*=1..29) to assess age-, reporter- and trait-specific variation in these polygenic association effects as explained by the fixed effect meta-regression parameters $\theta_{age}, \theta_{rater,}$ $\theta_{trait}$ and $\theta_{cohort}$. We studied a meta-regression model as given by

$\beta_{i}=\theta_{0}+\theta_{age}x_{{age}_{i}}+ \theta_{rater}x_{{rater}_{i}}+\theta_{trait}x_{{trait}_{i}}+\theta_{cohort}x_{{cohort}_{i}} + v_{i/k}$ [9]

including a random intercept ($v_{i/k}$) accounting for within-sample relatedness among social scores nested within each cohort (ALSPAC and TEDS, k=2), a fixed effect $\theta_{cohort}$for $x_{{cohort}_{i}}$ the dummy-coded indicator of assessment within ALSPAC (0) versus TEDS (1) for PRS effect *i*, a fixed effect $\theta_{age}$for $x_{{age}_{i}}$ the median age for the social score associated with PRS effect i, a fixed effect $\theta_{rater}$for $x_{{rater}_{i}}$ the dummy-coded parent (0) versus teacher-report (1) for the social score associated with PRS effect i, and a fixed effect $\theta_{trait}$ for $x_{{trait}_{i}}$the dummy-coded low-prosociality (0) versus peer-problems (1) score related to PRS effect *i*, assuming that $v_{i/k}\sim N(0,\tau^{2})$. $\tau^{2}$ denotes the amount of residual heterogeneity among the true effects^36^. Note that we combined PRS effects across peer problems and low prosociality as each was assessed with a 5-item subscale of the SDQ and recorded as item counts. We first established whether cohort-specific fixed effects $\theta_{cohort}$contributed to the model. If not, we dropped it from further modelling. Next, we identified for each disorder the most parsimonious model, confirmed by likelihood-ratio tests at *P*>0.05 against a full model that included fixed effects for age, reporter, and trait, and applied Cochran’s Q test to assess residual heterogeneity. For sensitivity analyses, we also compared combinations of univariate PRS(C+T) effects with combinations of univariate PRS-CS effects, in ALSPAC only. Here, fixed effects for cohort ($\theta_{cohort}$) were not included in the meta-regression.

We accounted for interrelatedness among PRS effects within each cohort using an approach that is analogous to models that account for correlated phylogenetic histories implementing a derived variance covariance matrix^37^. This variance covariance matrix was given by $V =SV_{pheno}S$ with $S$ being a diagonal matrix with the standard errors (SE) of PRS effect estimates

$S=\left[ \begin{matrix} {SE}_{PRS 1} & & \\ & {SE}_{PRS 1} & \\ & & \begin{matrix} \ddots& \\ & {SE}_{PRS 29} \end{matrix} \end{matrix} \right]$ [10]

and $V_{pheno}$ being the Pearson’s phenotypic correlation matrix of the social scores:

$V_{pheno}=\left[ \begin{aligned} \begin{matrix} 1 & {corr}_{1 2} & \cdots\\ {corr}_{2 1} & 1 & \\ \vdots& \vdots& \ddots\end{matrix} \\ \begin{matrix} {corr}_{29 1} & {corr}_{29 2} & \ldots\end{matrix} \end{aligned}\begin{matrix} {corr}_{1 29} \\ \\ \begin{matrix} \vdots\\ 1 \end{matrix} \end{matrix} \right]$. [11]

R^2^ is reported as the ratio of explained variance to total variance in univariate PRS effects β_i_.

## *Gene ontology biological pathway-based PRS analyses in ALSPAC*

For social traits with the strongest meta-analytically predicted PRS associations in ALSPAC, we conducted pathway-based PRS analyses using PRSet^38^. Defining for each disorder a baseline at *P*_T_≤0.1, we constructed subsets of pathway-PRS for 7,481 gene sets based on gene ontology biological pathways. Thus, we calculated ADHD-, ASD-, BP-, MD- and schizophrenia-based pathway-PRS at *P*_T_ ≤ 0.1 for 7,481 gene ontology gene sets (MSigDB database, v7.4). Additionally, we calculated for each disorder baseline-PRS based on all markers across all gene sets passing *P*_T_ ≤ 0.1. As described for the PRS(C+T) approach above, we studied high-quality genome-wide imputed SNPs with clumping thresholds of LD-r^2^>0.25 and ±500 kb. Note, however, that in PRSet, clumping is conducted for each gene set separately. Therefore, SNP numbers at *P*_T_ ≤0.1 for baseline-PRS in PRSet are marginally lower compared to those obtained with PRS(C+T) in PLINK. For each pathway-PRS and baseline-PRS, we estimated the association between polygenic risk for mental disorder and social behaviour in ALSPAC and the corresponding ΔMc Fadden’s R^2^, applying a negative binomial regression model as described for the PRS(C+T) approach above. To control for inflated type I error, we screened for pathway-PRS that reached the same strength of association as baseline-PRS and passed the multiple-testing threshold in ALSPAC (*P*_pathway_≤*P*_baseline_≤0.001).

## *Web resources*

ALSPAC data dictionary: <http://www.bris.ac.uk/alspac/researchers/data-access/data-dictionary/>

ALSPAC variable catalogue: http://www.bristol.ac.uk/alspac/researchers/access/

PGC: http://www.med.unc.edu/pgc

iPSYCH: http://ipsych.au.dk

UKBB: https://www.ukbiobank.ac.uk/

PLINK: https://www.cog-genomics.org/plink2

PRS-CS: https://github.com/getian107/PRScs

PRSet: https://www.prsice.info/prset_detail/

MSigDB: https://www.gsea-msigdb.org/gsea/msigdb/

HRC: http://www.haplotype-reference-consortium.org/

SANGER IMPUTATION SERVER: https://imputation.sanger.ac.uk/

LDSC: https://github.com/bulik/ldsc

R: https://www.r-project.org/

METAFOR: http://www.metafor-project.org/doku.php

# **Supplementary Figures**

**
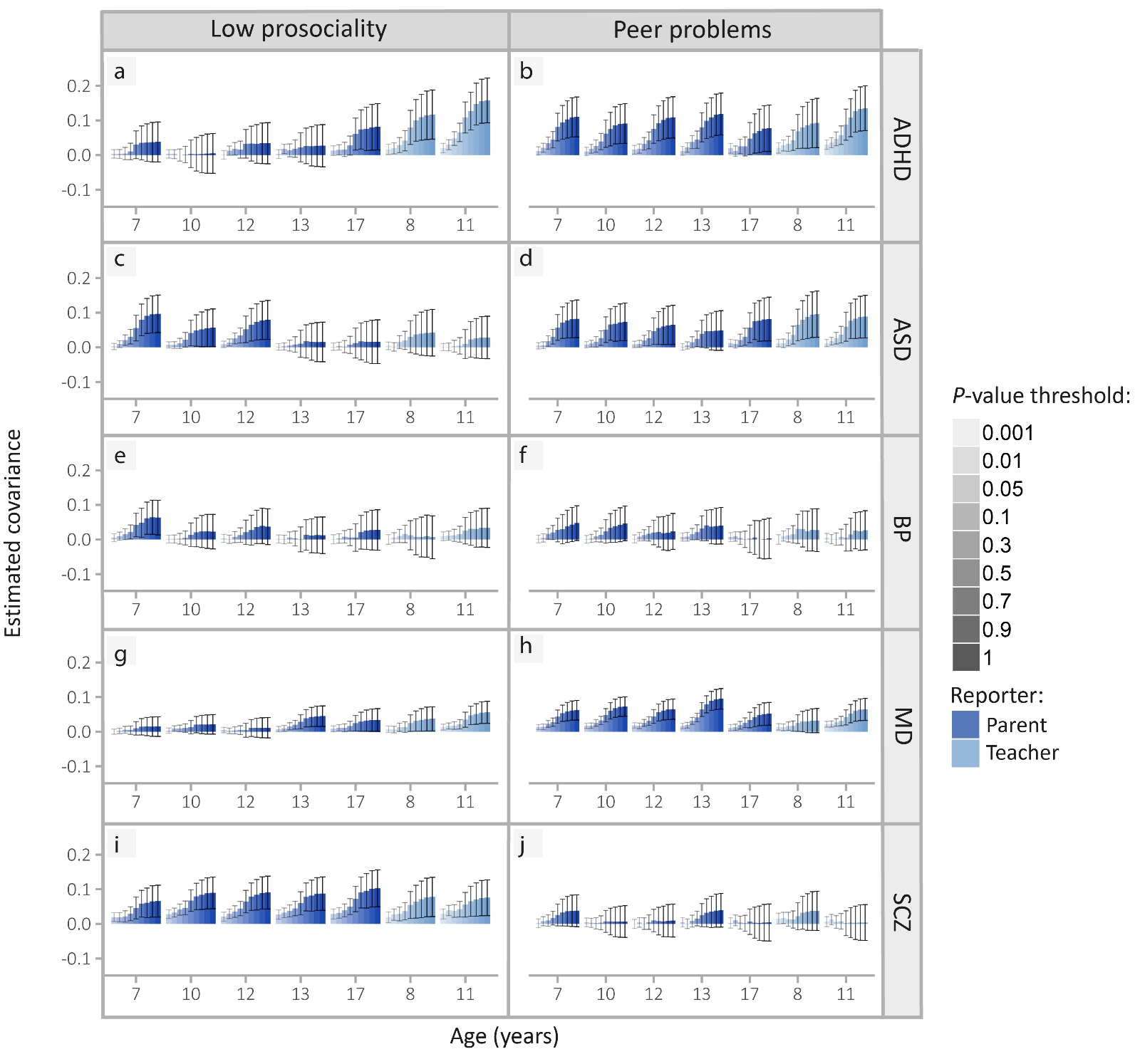
**

**Supplementary Figure 1:** Estimation of trait-disorder covariance based on mental disorder PRS(C+T) constructed across different P-value thresholds in the ALSPAC discovery samples using low-prosociality and peer-problem scores reported by parents or teachers at the age of 7 to 17 years. Here, the proportion of markers with no effect on the training set (pi0) was set to 0.95.

Analyses were conducted using the R software package avengeme^35^ accounting for the heritability of the discovery sample, the sample sizes of the discovery and the target sample, and the number of genetic variants used to construct PRS. Bars represent 95%-confidence intervals.

ADHD - Attention-deficit/hyperactivity disorder; ALSPAC - Avon Longitudinal study of Parents and Children; ASD - Autism spectrum disorders; BP - Bipolar disorder; C+T - clumping and thresholding; MD- Major depression; PRS-Polygenic risk scores; SCZ - Schizophrenia


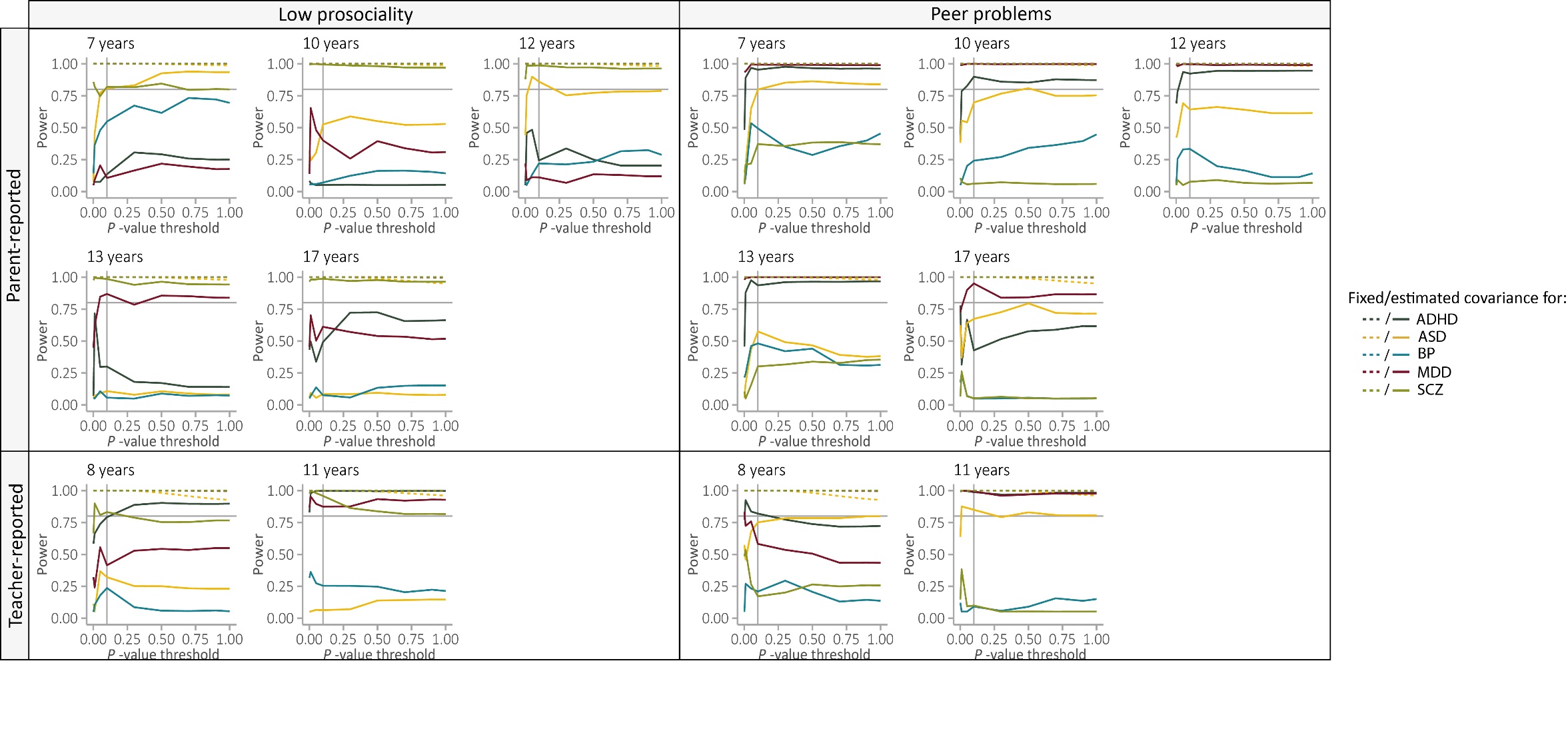


**Supplementary Figure 2:** Power-analysis of mental disorder PRS(C+T) constructed across different P-value thresholds in the ALSPAC discovery samples using low-prosociality and peer-problem scores reported by parents or teachers at the age of 7 to 17 years. Here, the proportion of markers with no effect on the training set (pi0) was set to 0.95.

Analyses were conducted using the R software package avengeme^35^ accounting for the heritability of the discovery sample, the sample sizes of the discovery and the target sample, and the number of genetic variants used to construct PRS.

ADHD - Attention-deficit/hyperactivity disorder; ALSPAC - Avon Longitudinal study of Parents and Children; ASD - Autism spectrum disorders; BP - Bipolar disorder; C+T - clumping and thresholding; MD- Major depression; PRS-Polygenic risk scores; SCZ – Schizophrenia

**
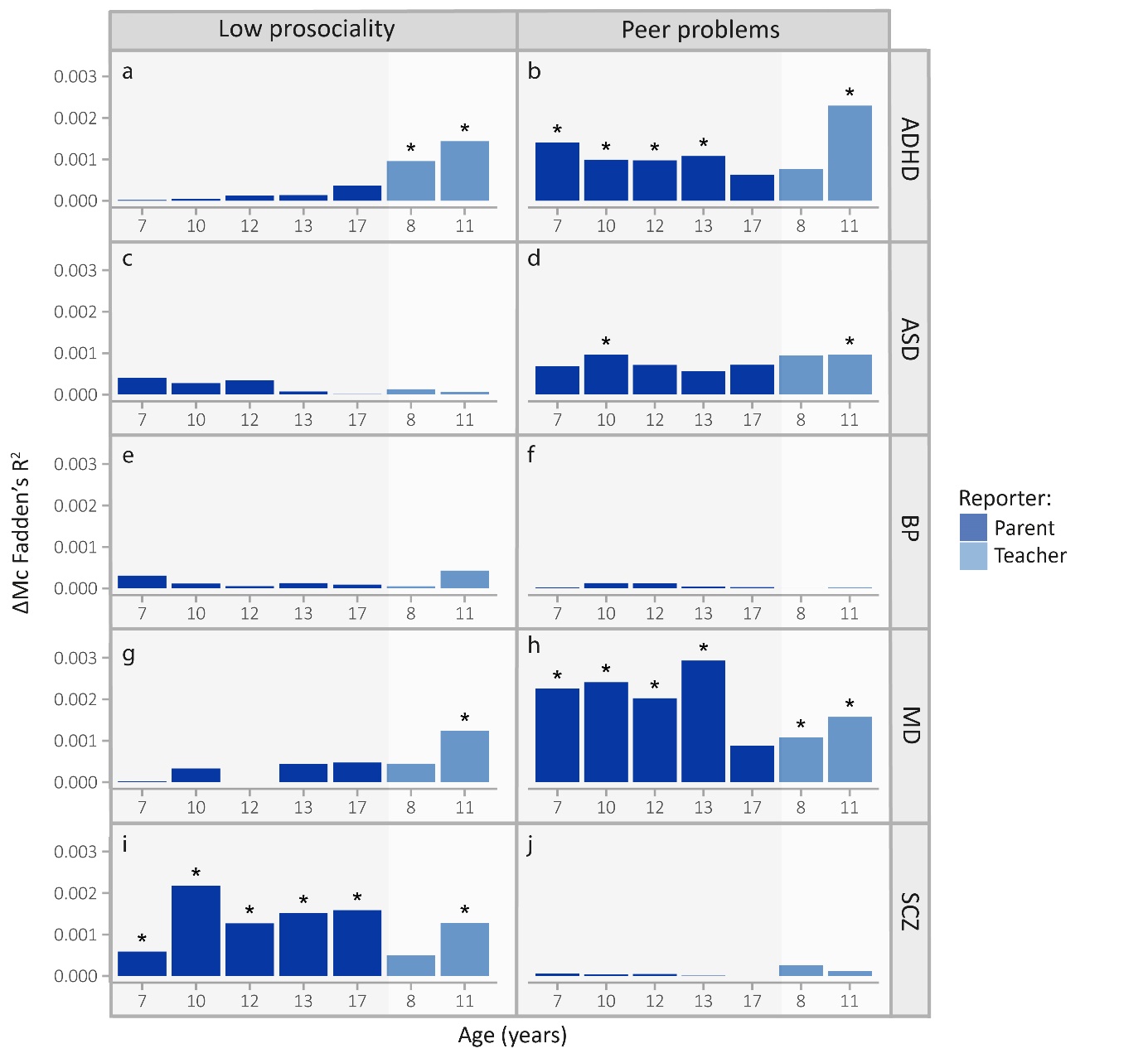
**

**Supplementary Figure 3:** Association between PRS (PRS-CS) for mental disorder and social behaviour in ALSPAC.

ΔMcFadden’s-R^2^ is shown for the prediction of low-prosociality and peer-problem scores by ADHD-PRS (a, b), ASD-PRS (c, d), BP-PRS (e,f), MD-PRS (g, h), SCZ-PRS (i, j).

Mental disorder samples (ADHD-PGC/iPSYCH, ASD-PGC/iPSYCH, BP-PGC, MD-PGC/UKBB, and SCZ-PGC) were used to construct Z-standardised PRS (PRS-CS) in ALSPAC (ADHD-PRS, ASD-PRS, BP-PRS, MD-PRS, and SCZ-PRS). Association analyses with social behaviour (low prosociality and peer problems) were conducted using negative binomial regression (non-adjusted for cross-disorder PRS effects; multiple-testing corrected *P*-value: **P*≤0.001).

ADHD - Attention-deficit/hyperactivity disorder; ALSPAC - Avon Longitudinal study of Parents and Children; ASD - Autism spectrum disorders; BP - Bipolar disorder; iPSYCH - Lundbeck Foundation Initiative for Integrative Psychiatric Research; MD - Major depression; PGC - Psychiatric Genomics consortium; PRS - Polygenic risk scores; SCZ - Schizophrenia

Low-prosociality and peer-problem scores were assessed using the Strengths-and-Difficulties questionnaire.


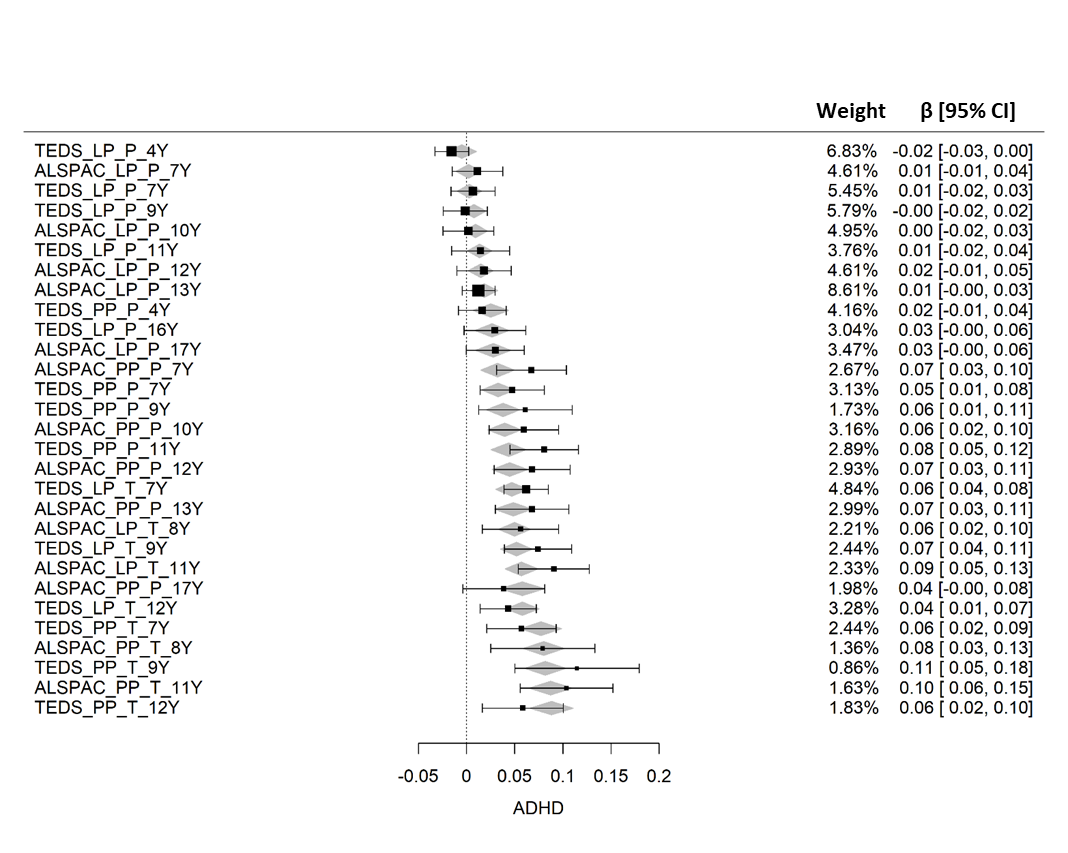


**Supplementary Figure 4:** Forest plot showing both univariate ADHD-PRS effects (β) on low-prosocialty and peer-problem scores (black squares) and predicted ADHD-PRS effects ($\hat{\beta}$; grey diamonds) using the most-parsimonious mixed-effects meta-regression model with respective 95% CI bands.

Univariate PRS(C+T) association effects (at P_T_≤0.1) for ADHD risk on social behaviour (negative binominal model non-adjusted for cross-disorder PRS effects) were combined across 29 social symptoms (14 ALSPAC-based + 15 TEDS-based) using mixed-effects meta-regressions, one for each disorder PRS, accounting for phenotypic correlations between social symptoms. The most-parsimonious predictors for heterogeneity in ADHD-PRS effects included age (years), reporter (parent vs teacher), and trait (low prosociality vs peer problems).

Low-prosociality and peer-problem scores were assessed using the Strengths-and-Difficulties questionnaire.

ADHD - Attention-deficit/hyperactivity disorder; ALSPAC - Avon Longitudinal study of Parents and Children; CI - Confidence interval; C+T - clumping and thresholding; LP - Low prosociality; P - Parent-report; PP - Peer problems; PRS - Polygenic risk scores; T - Teacher-report; TEDS - Twins Early Development Study; Y - Age in years


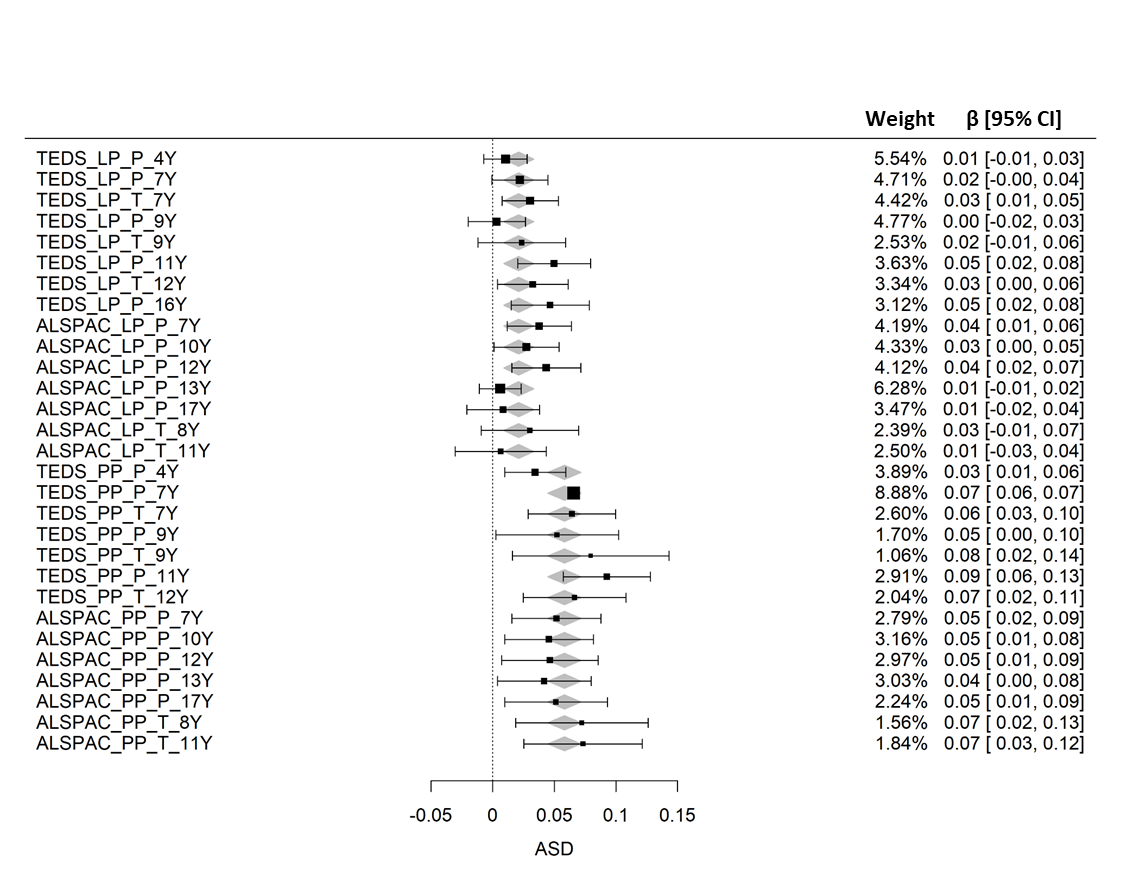


**Supplementary Figure 5:** Forest plot showing both univariate ASD-PRS effects (β) on low-prosocialty and peer-problem scores (black squares) and predicted ASD-PRS effects ($\hat{\beta}$; grey diamonds) using the most-parsimonious mixed-effects meta-regression model with respective 95% CI bands.

Univariate PRS(C+T) association effects (at P_T_≤0.1) for ASD risk on social behaviour (negative binominal model non-adjusted for cross-disorder PRS effects) were combined across 29 social symptoms (14 ALSPAC-based + 15 TEDS-based) using mixed-effects meta-regressions, one for each disorder PRS, accounting for phenotypic correlations between social symptoms. The most-parsimonious predictor for heterogeneity in ASD-PRS effects included trait (low prosociality vs peer problems).

Low-prosociality and peer-problem scores were assessed using the Strengths-and-Difficulties questionnaire.

ASD - Autism spectrum disorders; ALSPAC - Avon Longitudinal study of Parents and Children; CI - Confidence interval; C+T - clumping and thresholding; LP - Low prosociality; P - Parent-report; PP - Peer problems; PRS-Polygenic risk scores; T - Teacher-report; TEDS - Twins Early Development Study; Y - Age in years

**
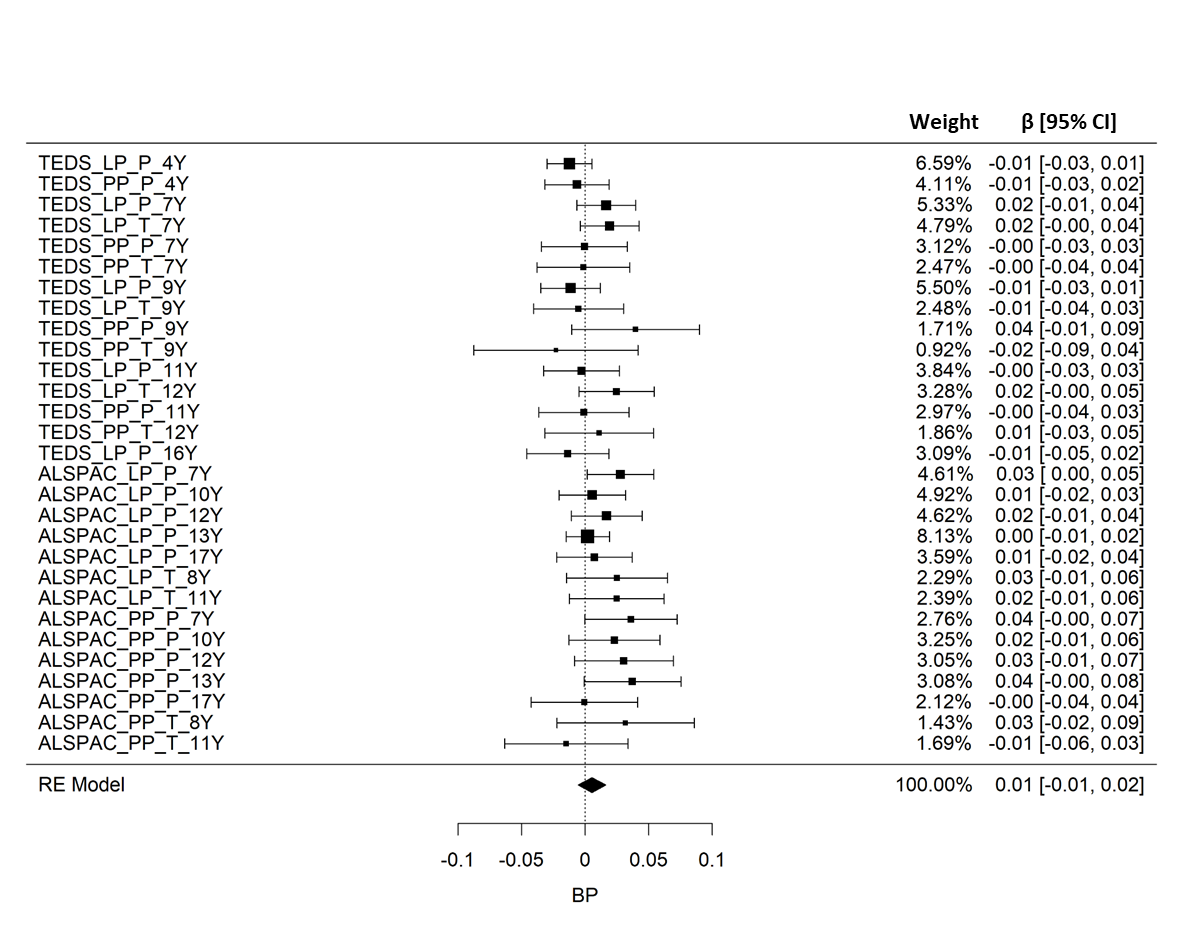
**

**Supplementary Figure 6:** Forest plot showing both univariate BP-PRS effects (β) on low-prosocialty and peer-problem scores (black squares) and predicted BP-PRS effect ($\hat{\beta}$; black diamond) using the most-parsimonious mixed-effects meta-regression model with respective 95% CI bands.

Univariate PRS(C+T) association effects (at P_T_≤0.1) for BP risk on social behaviour (negative binominal model non-adjusted for cross-disorder PRS effects) were combined across 29 social symptoms (14 ALSPAC-based + 15 TEDS-based) using mixed-effects meta-regressions, one for each disorder PRS, accounting for phenotypic correlations between social symptoms. The most-parsimonious model for BP-PRS effects was the intercept model.

Low-prosociality and peer-problem scores were assessed using the Strengths-and-Difficulties questionnaire.

ALSPAC - Avon Longitudinal study of Parents and Children; BP - Bipolar disorder; CI - Confidence interval; C+T - clumping and thresholding; LP - Low prosociality; P - Parent-report; PP - Peer problems; PRS -Polygenic risk scores; T - Teacher-report; TEDS - Twins Early Development Study; Y - Age in years


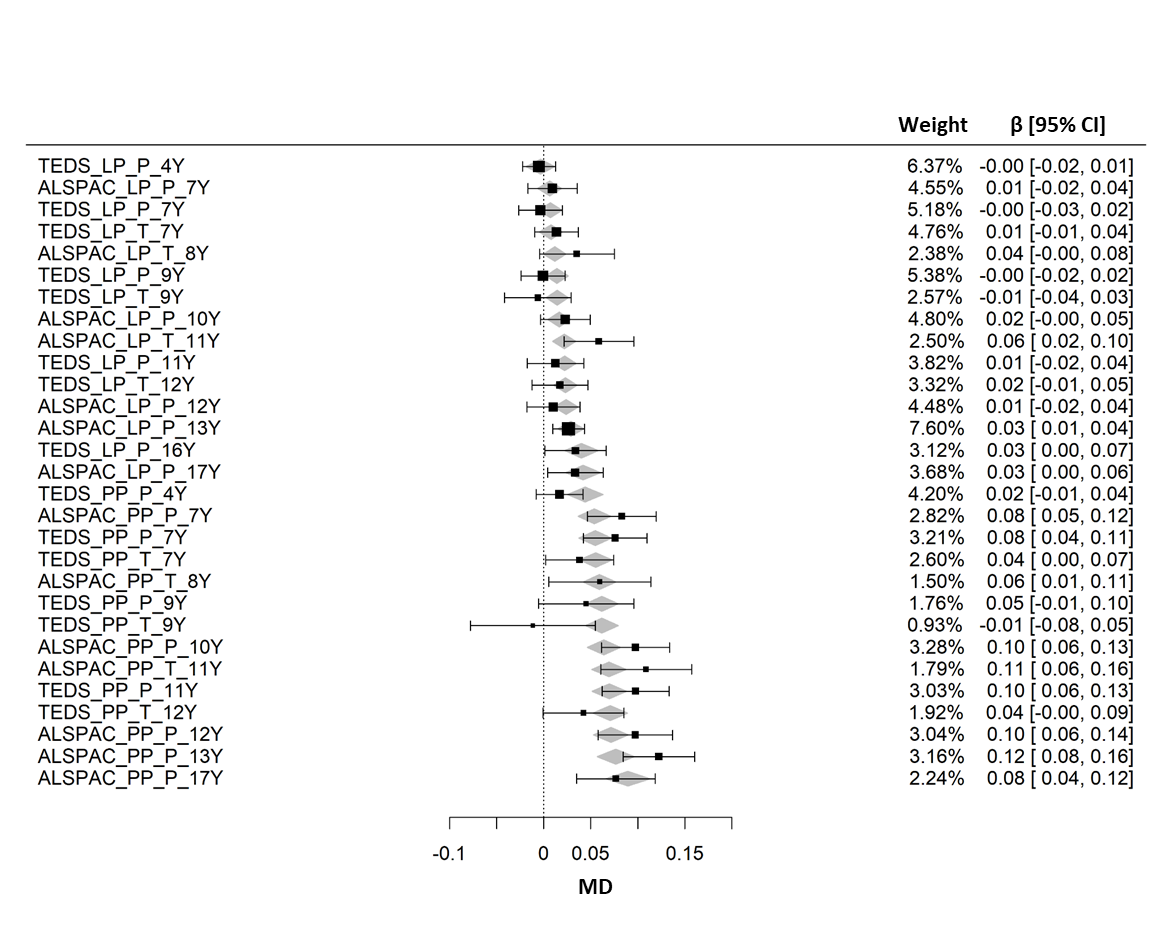


**Supplementary Figure 7:** Forest plot showing both univariate MD-PRS effects (β) on low-prosocialty and peer-problem scores (black squares) and predicted MD-PRS effects ($\hat{\beta}$; grey diamonds) using the most-parsimonious mixed-effects meta-regression model with respective 95% CI bands.

Univariate PRS(C+T) association effects (at P_T_≤0.1) for MD risk on social behaviour (negative binominal model non-adjusted for cross-disorder PRS effects) were combined across 29 social symptoms (14 ALSPAC-based + 15 TEDS-based) using mixed-effects meta-regressions, one for each disorder PRS, accounting for phenotypic correlations between social symptoms. The most-parsimonious predictor for heterogeneity in MD-PRS effects included age (years) and trait (low prosociality vs peer problems).

Low-prosociality and peer-problem scores were assessed using the Strengths-and-Difficulties questionnaire.

ALSPAC - Avon Longitudinal study of Parents and Children; CI - Confidence interval; C+T - clumping and thresholding; LP - Low prosociality; MD - Major depression; P- Parent-report; PP - Peer problems; PRS -Polygenic risk scores; T - Teacher-report; TEDS - Twins Early Development Study; Y - Age in years


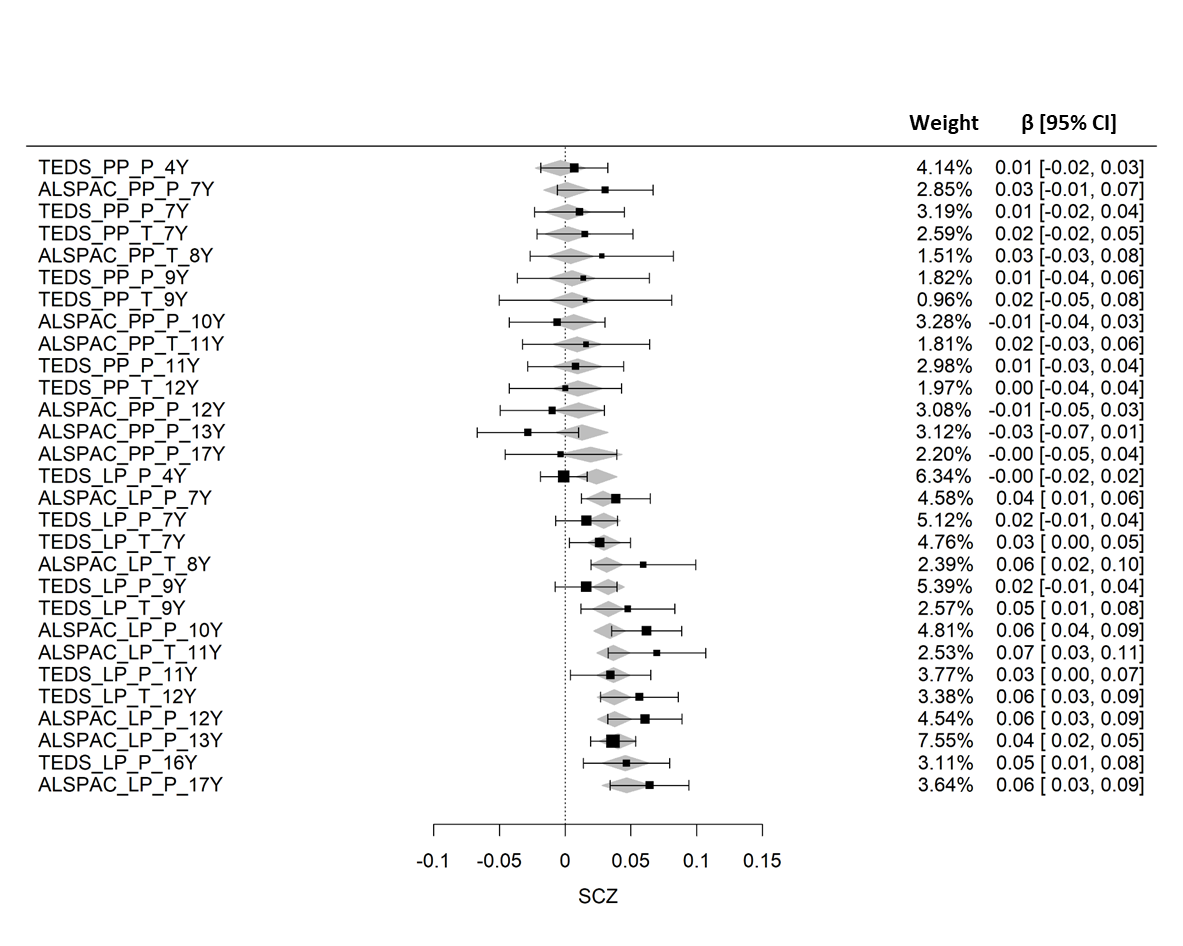


**Supplementary Figure 8:** Forest plot showing both univariate schizophrenia-PRS effects (β) on low-prosocialty and peer-problem scores (black squares) and predicted schizophrenia-PRS effects ($\hat{\beta}$; grey diamonds) using the most-parsimonious mixed-effects meta-regression model with respective 95% CI bands.

Univariate PRS(C+T) association effects (at P_T_≤0.1) for schizophrenia risk on social behaviour (negative binominal model non-adjusted for cross-disorder PRS effects) were combined across 29 social symptoms (14 ALSPAC-based + 15 TEDS-based) using mixed-effects meta-regressions, one for each disorder PRS, accounting for phenotypic correlations between social symptoms. The most-parsimonious predictor for heterogeneity in schizophrenia-PRS effects included age (years) and trait (low prosociality vs peer problems).

Low-prosociality and peer-problem scores were assessed using the Strengths-and-Difficulties questionnaire.

ALSPAC - Avon Longitudinal study of Parents and Children; CI - Confidence interval; C+T - clumping and thresholding; LP - Low prosociality; P - Parent-report; PP - Peer problems; PRS - Polygenic risk scores; SCZ - Schizophrenia; T - Teacher-report; TEDS - Twins Early Development Study; Y - Age in years

# **Supplementary References**

1. Demontis D, Walters RK, Martin J, Mattheisen M, Als TD, Agerbo E, *et al.* Discovery of the first genome-wide significant risk loci for attention deficit/hyperactivity disorder. *Nat Genet* 2019; **51**: 63–75.

2. Pedersen CB, Bybjerg-Grauholm J, Pedersen MG, Grove J, Agerbo E, Bækvad-Hansen M, *et al.* The iPSYCH2012 case–cohort sample: new directions for unravelling genetic and environmental architectures of severe mental disorders. *Mol Psychiatry* 2018; **23**: 6–14.

3. Mors O, Perto GP, Mortensen PB. The Danish Psychiatric Central Research Register. *Scand J Public Health* 2011; **39**: 54–7.

4. Grove J, Ripke S, Als TD, Mattheisen M, Walters RK, Won H, *et al.* Identification of common genetic risk variants for autism spectrum disorder. *Nat Genet* 2019; **51**: 431–44.

5. Anney RJ, Ripke S, Anttila V, Grove J, Holmans P, Huang H, *et al.* Meta-analysis of GWAS of over 16,000 individuals with autism spectrum disorder highlights a novel locus at 10q24. 32 and a significant overlap with schizophrenia. *Mol Autism* 2017; **8**: 1–17.

6. Cross-Disorder Group of the Psychiatric Genomics Consortium. Identification of risk loci with shared effects on five major psychiatric disorders: a genome-wide analysis. *Lancet* 2013; **381**: 1371–9.

7. Stahl EA, Breen G, Forstner AJ, McQuillin A, Ripke S, Trubetskoy V, *et al.* Genome-wide association study identifies 30 loci associated with bipolar disorder. *Nat Genet* 2019; **51**: 793–803.

8. Howard DM, Adams MJ, Clarke T-K, Hafferty JD, Gibson J, Shirali M, *et al.* Genome-wide meta-analysis of depression identifies 102 independent variants and highlights the importance of the prefrontal brain regions. *Nat Neurosci* 2019; **22**: 343–52.

9. Wray NR, Ripke S, Mattheisen M, Trzaskowski M, Byrne EM, Abdellaoui A, *et al.* Genome-wide association analyses identify 44 risk variants and refine the genetic architecture of major depression. *Nat Genet* 2018; **50**: 668–81.

10. Howard DM, Adams MJ, Shirali M, Clarke T-K, Marioni RE, Davies G, *et al.* Genome-wide association study of depression phenotypes in UK Biobank identifies variants in excitatory synaptic pathways. *Nat Commun* 2018; **9**: 1–10.

11. Smith DJ, Nicholl BI, Cullen B, Martin D, Ul-Haq Z, Evans J, *et al.* Prevalence and Characteristics of Probable Major Depression and Bipolar Disorder within UK Biobank: Cross-Sectional Study of 172,751 Participants. *PLOS ONE* 2013; **8**: e75362.

12. Schizophrenia Working Group of the Psychiatric Genomics Consortium. Biological insights from 108 schizophrenia-associated genetic loci. *Nature* 2014; **511**: 421–7.

13. A map of human genome variation from population-scale sequencing. *Nature* 2010; **467**: 1061–73.

14. A global reference for human genetic variation. *Nature* 2015; **526**: 68–74.

15. McCarthy S, Das S, Kretzschmar W, Delaneau O, Wood AR, Teumer A, *et al.* A reference panel of 64,976 haplotypes for genotype imputation. *Nat Genet* 2016; **48**: 1279–83.

16. Bycroft C, Freeman C, Petkova D, Band G, Elliott LT, Sharp K, *et al.* Genome-wide genetic data on ~500,000 UK Biobank participants. *bioRxiv* 2017; 166298.

17. Fraser A, Macdonald-Wallis C, Tilling K, Boyd A, Golding J, Davey Smith G, *et al.* Cohort Profile: the Avon Longitudinal Study of Parents and Children: ALSPAC mothers cohort. *Int J Epidemiol* 2013; **42**: 97–110.

18. Boyd A, Golding J, Macleod J, Lawlor DA, Fraser A, Henderson J, *et al.* Cohort Profile: the ’children of the 90s’--the index offspring of the Avon Longitudinal Study of Parents and Children. *Int J Epidemiol* 2013; **42**: 111–27.

19. Goodman R. The Strengths and Difficulties Questionnaire: a research note. *J Child Psychol Psychiatry* 1997; **38**: 581–6.

20. Verhoef E, Demontis D, Burgess S, Shapland CY, Dale PS, Okbay A, *et al.* Disentangling polygenic associations between attention-deficit/hyperactivity disorder, educational attainment, literacy and language. *Transl Psychiatry* 2019; **9**: 1–12.

21. Selzam S, McAdams TA, Coleman JRI, Carnell S, O’Reilly PF, Plomin R, *et al.* Evidence for gene-environment correlation in child feeding: Links between common genetic variation for BMI in children and parental feeding practices. *PLOS Genet* 2018; **14**: e1007757.

22. Gelman A, Hill J. *Data analysis using regression and multilevel/hierarchical models*. Cambridge university press: 2006.

23. Gardner W, Mulvey EP, Shaw EC. Regression analyses of counts and rates: Poisson, overdispersed Poisson, and negative binomial models. *Psychol Bull* 1995; **118**: 392–404.

24. Faraway JJ. *Extending the linear model with R: generalized linear, mixed effects and nonparametric regression models*. CRC press: 2016.

25. Roshchupkin GV, Adams H, Vernooij M, Hofman A, Van Duijn C, Ikram M, *et al.* HASE: Framework for efficient high-dimensional association analyses. *Sci Rep* 2016; **6**: 1–8.

26. Bulik-Sullivan BK, Loh P-R, Finucane HK, Ripke S, Yang J, Patterson N, *et al.* LD Score regression distinguishes confounding from polygenicity in genome-wide association studies. *Nat Genet* 2015; **47**: 291–5.

27. Devlin B, Roeder K. Genomic Control for Association Studies. *Biometrics* 1999; **55**: 997–1004.

28. Polanczyk GV, Salum GA, Sugaya LS, Caye A, Rohde LA. Annual Research Review: A meta‐analysis of the worldwide prevalence of mental disorders in children and adolescents. *J Child Psychol Psychiatry* 2015; **56**: 345–65.

29. Merikangas KR, Jin R, He J-P, Kessler RC, Lee S, Sampson NA, *et al.* Prevalence and correlates of bipolar spectrum disorder in the world mental health survey initiative. *Arch Gen Psychiatry* 2011; **68**: 241–51.

30. Kupfer DJ, Frank E, Phillips ML. Major depressive disorder: new clinical, neurobiological, and treatment perspectives. *Lancet* 2012; **379**: 1045–55.

31. Owen MJ, Sawa A, Mortensen PB. Schizophrenia. *Lancet* 2016; **388**: 86–97.

32. Purcell S, Neale B, Todd-Brown K, Thomas L, Ferreira MA, Bender D, *et al.* PLINK: a tool set for whole-genome association and population-based linkage analyses. *Am J Hum Genet* 2007; **81**: 559–75.

33. Wray NR, Lee SH, Mehta D, Vinkhuyzen AAE, Dudbridge F, Middeldorp CM. Research review: polygenic methods and their application to psychiatric traits. *J Child Psychol Psychiatry* 2014; **55**: 1068–87.

34. Ge T, Chen C-Y, Ni Y, Feng Y-CA, Smoller JW. Polygenic prediction via Bayesian regression and continuous shrinkage priors. *Nat Commun* 2019; **10**: 1776.

35. Dudbridge F. Power and Predictive Accuracy of Polygenic Risk Scores. *PLOS Genet* 2013; **9**: e1003348.

36. Viechtbauer W. Conducting meta-analyses in R with the metafor package. *J Stat Softw* 2010; **36**: 1–48.

37. Lajeunesse MJ. Meta-analysis and the comparative phylogenetic method. *Am Nat* 2009; **174**: 369–81.

38. Choi SW, Garcia-Gonzalez J, Ruan Y, Man Wu H, Johnson J, Hoggart C, *et al.* The power of pathway-based polygenic risk scores. *Res Sq* 2021;
